# Trajectories in chronic disease accrual and mortality across the lifespan in Wales, UK (2005–2019), by area deprivation profile: linked electronic health records cohort study on 965,905 individuals

**DOI:** 10.1016/j.lanepe.2023.100687

**Published:** 2023-07-17

**Authors:** Jane Lyons, Ashley Akbari, Keith R. Abrams, Amaya Azcoaga Lorenzo, Thamer Ba Dhafari, James Chess, Spiros Denaxas, Richard Fry, Chris P. Gale, John Gallacher, Lucy J. Griffiths, Bruce Guthrie, Marlous Hall, Farideh Jalali-najafabadi, Ann John, Clare MacRae, Colin McCowan, Niels Peek, Dermot O’Reilly, James Rafferty, Ronan A. Lyons, Rhiannon K. Owen

**Affiliations:** a Population Data Science, Swansea University Medical School, Faculty of Medicine, Health & Life Science, Swansea University, Swansea, Wales, UK; b Department of Statistics, University of Warwick, Coventry, UK; c Centre for Health Economics, University of York, York, UK; d Instituto Investigación Sanitaria Fundación Jimenez Diaz, Madrid, Spain; e School of Medicine, University of St Andrews, St Andrews, UK; f Division of Informatics, Imaging and Data Science, School of Health Sciences, University of Manchester, Manchester, UK; g Swansea Bay Health Board, Morriston Hospital, Swansea, Wales, UK; h Institute of Health Informatics, University College London, London, UK; i School of Medicine, University of Leeds, Leeds, UK; j Dementias Platform UK, Department of Psychiatry, University of Oxford, Oxford, UK; k Advanced Care Research Centre, Usher Institute, University of Edinburgh, Edinburgh, UK; l Leeds Institute of Cardiovascular and Metabolic Medicine and Leeds Institute for Data Analytics, University of Leeds, Leeds, UK; m Centre for Genetics and Genomics Versus Arthritis, Centre for Musculoskeletal Research, Faculty of Biology, Medicine and Health, Manchester Academic Health Science Centre, University of Manchester, Manchester, UK; n School of Medicine, Dentistry and Biomedical Sciences, Queen’s University Belfast, Belfast, UK; o Swansea Trials Unit, Swansea University Medical School, Faculty of Medicine, Health & Life Science, Swansea University, Swansea, Wales, UK

**Keywords:** Chronic disease, Mortality, Disease trajectories, Population-wide, Health equity

## Abstract

**Background:**

Understanding and quantifying the differences in disease development in different socioeconomic groups of people across the lifespan is important for planning healthcare and preventive services. The study aimed to measure chronic disease accrual, and examine the differences in time to individual morbidities, multimorbidity, and mortality between socioeconomic groups in Wales, UK.

**Methods:**

Population-wide electronic linked cohort study, following Welsh residents for up to 20 years (2000–2019). Chronic disease diagnoses were obtained from general practice and hospitalisation records using the CALIBER disease phenotype register. Multi-state models were used to examine trajectories of accrual of 132 diseases and mortality, adjusted for sex, age and area-level deprivation. Restricted mean survival time was calculated to measure time spent free of chronic disease(s) or mortality between socioeconomic groups.

**Findings:**

In total, 965,905 individuals aged 5–104 were included, from a possible 2.9 m individuals following a 5-year clearance period, with an average follow-up of 13.2 years (12.7 million person-years). Some 673,189 (69.7%) individuals developed at least one chronic disease or died within the study period. From ages 10 years upwards, the individuals living in the most deprived areas consistently experienced reduced time between health states, demonstrating accelerated transitions to first and subsequent morbidities and death compared to their demographic equivalent living in the least deprived areas. The largest difference were observed in 10 and 20 year old males developing multimorbidity (−0.45 years (99% CI: −0.45, −0.44)) and in 70 year old males dying after developing multimorbidity (−1.98 years (99% CI: −2.01, −1.95)).

**Interpretation:**

This study adds to the existing literature on health inequalities by demonstrating that individuals living in more deprived areas consistently experience accelerated time to diagnosis of chronic disease and death across all ages, accounting for competing risks.

**Funding:**

10.13039/501100000265UK Medical Research Council, Health Data Research UK, and Administrative Data Research Wales.

**Research in context**

**Evidence before this study**

We searched PubMed without language restrictions for longitudinal or population-based published studies between database inception and 14th April 2023 that utilised multi-state models to examine disease trajectories over time. The search terms were (((multi-state) OR (multistate)) AND ((trajectories) OR (trajectory) OR (transition) OR (transitions)) AND ((morbidity) OR (multimorbidity) OR (multi-morbidity) OR (disease)) AND ((longitudinal) OR (population))). Several studies have used multi-state models to examine transitioning between health states in developing chronic diseases and multimorbidity, however, existing studies have focused on disease-specific outcomes, or accumulation of a limited number of diseases, were based on adults only, or selected cohorts rather than across the lifespan, or did not focus on health disparities.

**Added value of this study**

To our knowledge, this study is the first to examine population-wide, long-term follow-up to measure the accrual of multiple chronic diseases and mortality, having included a disease clearance period and appropriately accounted for competing risks, in producing direct comparisons of trajectories of health outcomes across different socioeconomic groups over a period of two decades. This study contributes to the evidence base demonstrating an acceleration in the accumulation of chronic morbidity and multimorbidity and premature mortality in individuals living in deprived areas across all age groups. Additionally, this study provides evidence of a disparity in time spent free of chronic disease(s) and mortality between males and females of the same age and deprivation. Females experience an accelerated accrual of chronic conditions compared to males, whereas males experience accelerated time to death compared to females.

**Implications of all the available evidence**

Our findings provide important evidence with both clinical and social policy implications. We provide quantifiable and comparable evidence of chronic disease and mortality inequity across all ages and sexes in Wales, UK. Our study adds to the existing literature on health inequalities by demonstrating that individuals living in more deprived areas consistently experience accelerated time to chronic disease diagnosis and death across all ages, accounting for competing risks.

This methodology could be used to evaluate interventions designed to slow down disease accrual with findings being used to inform health and social policy and clinical practice.

## Introduction

Reducing health inequalities within and between countries is a global priority to improve human life.^1^ Previous research has shown that living in poverty and worse socioeconomic circumstances are associated with poor health.^2^^,^^3^ Being more socioeconomically deprived has shown to be associated with increased morbidity, multimorbidity, and mortality.^4^^,^^5^ Chronic morbidity is defined as the existence of one chronic health condition in an individual, whereas multimorbidity is defined as the presence of two or more concurrent chronic health conditions.^6^ Chronic morbidity and multimorbidity are associated with reduced quality of life, increased healthcare utilisation, polypharmacy, and mortality.^7^, ^8^, ^9^ Despite being informative, previous multimorbidity studies have largely focused on the incidence or prevalence disease-specific outcomes, or health outcomes in adults and older individuals rather than transitions in disease states across the population or by deprivation.^10^, ^11^, ^12^, ^13^

Some studies have used research cohorts to follow up samples of the population or increasingly total population linked electronic health record studies.^14^ For example, the Danish Atlas of Disease Mortality study which included 1803 individual conditions in 7.9 M people, reported on incidence, age of onset, and mortality but not the transition from morbidity to multimorbidity or from multimorbidity to death.^15^

Better understanding of the timing and transition from a disease-free state to chronic morbidity, multi-morbidity and death is important for planning health system and targeting interventions. Multi-state models have been used to investigate transitions between health states in older people, disease or disorder-specific pathways across a limited number of conditions.^16^, ^17^, ^18^

The aim of this study was to use multi-state models to measure the accrual of 132 chronic diseases, appropriately accounting for competing risk of death, and examine the differences in trajectories of morbidity, multimorbidity, and mortality between socioeconomic groups in Wales, UK. We hypothesised that individuals living in more deprived areas will accrue diseases at a faster rate compared to more affluent areas, demonstrating the implication of socioeconomic inequity on accelerating morbidity and mortality.

## Methods

### Study design

In this observational, longitudinal, population-wide cohort study, we used the Wales Multimorbidity e-Cohort (WMC) held within the Secure Anonymised Information Linkage (SAIL) Databank which follows 2.9 million residents of Wales for up to 20 years from 2000–2019.^19^, ^20^, ^21^ Electronic health records (EHR) derived from primary and secondary care sources were used to capture disease diagnoses, with mortality data captured in the national death register. We utilised multi-state models to model trajectories of chronic disease accrual and death, adjusted for potential competing risk of death.^22^^,^^23^

### Data sources

This study used routinely collected anonymised, individual-level, population-scale health and demographic data held in the SAIL Databank to create the retrospective population-based individual-level linked WMC.^19^, ^20^, ^21^ The WMC contains linked demographic, primary and secondary healthcare, and mortality data on all individuals living in Wales on the 1st January 2000 with follow-up until first date of migration out of Wales/residency break, death, or the study endpoint on 31st December 2019.

Demographic data were obtained from the Welsh Demographic Service Dataset, containing administrative information and sociodemographic characteristics, including age, sex, and area-level deprivation for socioeconomic categorisation, for the resident population of Wales registered with a Welsh General Practice. Socioeconomic groups were formed by grouping individuals based on the Lower layer Super Output Area of residence into income deprivation quintiles using the Welsh Index of Multiple Deprivation (WIMD) version 2019 at study end.^24^ Primary healthcare data were obtained from the Welsh Longitudinal General Practice database which contains all general practice (GP) events for individuals registered to a SAIL providing GP. SAIL holds GP data for 83.4% of GPs across all health boards in Wales. Secondary healthcare data were obtained from the Patient Episode Database for Wales (PEDW) of all hospital admissions, inclusive of day cases, for all NHS Wales hospitals as well as hospital admissions for Welsh residents treated at NHS England hospitals. Disease diagnoses and dates of diagnoses were obtained from both GP and hospitalisation records using a comprehensive disease phenotype register, the CALIBER resource to map disease phenotypes to individual International Classification of Diseases-10, Office of Population Censuses and Surveys codes version-4, and Read codes V2.^25^ CALIBER conditions were reviewed by an expert in clinical coding (RAL) to categorise conditions into acute and chronic, with 132 chronic conditions, ranging from cardiovascular, respiratory, cancer to mental health, included (Supplementary Table S1).

The Annual District Death Extract from the Office for National Statistics was used to capture all deaths and dates of death that occurred over the study period for all Welsh residents.

### Participants

All individuals from the WMC, alive and living in Wales on 1st January 2005 with follow up until death, migration or study endpoint of 31st December 2019 were included. We excluded individuals who had any of the 132 chronic disease diagnoses between 1st January 2000 and 31st December 2004 to allow for a 5-year clearance, with individuals starting from a chronic disease-free (CDF) state and capturing first diagnosis from 1st January 2005 (Supplementary Figure S1). We conducted analysis to evaluate the impact of varying the clearance period from 0 to 10 years where none of the 132 disease diagnoses occurred (Supplementary Table S2) and used a 5-year clearance allowing for a 15-year follow-up, with sensitivity analyses using 1 and 10-year clearances. We restricted the study population to include individuals who were registered with a SAIL providing GP to capture all possible diagnoses regardless of healthcare setting.

### Outcome measures

The primary outcome measure was Restricted Mean Survival Time (RMST) which represents the average time free from the event (development of disease or death), and is measured as the average time (in years) since entry to the health state and transition to subsequent health states including single chronic morbidity, multi-morbidity (2+ chronic diseases), and death. The time to first chronic disease diagnosis (morbidity), time to second chronic disease diagnosis (multi-morbidity), or time to death in years were used as the timescale for transitions between health states. The difference in RMST between socioeconomic groups was used to examine the speed of transitions between states and subsequently demonstrating differences in time to developing a chronic disease(s) or dying.

### Statistical methods

This study utilised multi-state models to model trajectories of chronic disease accrual accounting for death as a competing risk.^22^ We applied a 4-state model (Supplementary Figure S2) with all individuals starting in a chronic disease-free state on 1st January 2005. From this state, individuals could transition to single chronic morbidity, multimorbidity, or death. The model allows simultaneous estimation of each trajectory via estimation of all possible forward transitions between health states.^23^

Cox Proportional Hazard regression models were fitted to adjust for covariate effects. Covariates included time-varying age, as a continuous variable, defined as the age an individual enters the health state, sex, and, area-level deprivation at study end.^24^ Individuals were censored, assuming independent censoring, on the first date of a break in Welsh residency, cohort end (31st December 2019), or date of death when death was a competing risk.^26^ The Schoenfeld test was used to assess proportional hazards assumption. Proportional hazards did not hold for the included covariates, therefore, RMST (with an upper limit of 15 years) was calculated for all possible transitions from entry to each health state to measure time spent, in years, between health states, and to compare between the socioeconomic areas.^27^^,^^28^ Covariates were either re-centred or the reference category modified to estimate baseline RMST as a measure of absolute risk for each of the covariate patterns.^29^^,^^30^ Sensitivity analyses were carried out with area-level deprivation measured at the study start (Supplementary Table S3). Additional sensitivity analyses were carried out to repeat the study based on a one and 10-year clearance period (Supplementary Tables S4 and S5). Analysis was performed using the mstate package in R version 4.1.3.^31^

All models were adjusted for age, sex, and area-level deprivation. Models were estimated for 10, 20, 30, 40, 50, 60, and 70 years of age, both for males and females, and for all deprivation quintiles to simulate the effect of population distribution change and to allow direct comparison of trajectories of disease accrual and death between the most and least deprived individuals in Wales, UK.

### Role of the funding source

The funders of the study had no role in the data collection, study design, analysis, interpretation of results, or writing of the manuscript.

## Results

In total, 965,905 individuals aged 5–104 on 1st January 2005 were included in this study, from a possible 2.9 m WMC individuals following a 5-year clearance period. The average follow-up time was 13.2 years equating to 12.7 million person-years. The median age at the study start was 33 years (interquartile range (IQR): 17–47), 55.5% were male, 20.5% were living in the most deprived quintile and 19.4% were in the least deprived quintile (Table 1). Overall, 624,823 individuals developed morbidity, 465,856 developed multimorbidity, and 56,485 died within the study period (Table 1, Supplementary Tables S6–S8).

**Table 1:** Summary statistics for each health state.

Health state and transition	Total	Median age (IQR)	Most deprived area		Second most deprived area		Middle deprivation area		Second least deprived area		Least deprived area		Male	Female
			Total (%)	Median age (IQR)	Total (%)	Median age (IQR)	Total (%)	Median age (IQR)	Total (%)	Median age (IQR)	Total (%)	Median age (IQR)	Total (%)	Total (%)
Baseline	965,905	33 (17–47)	197,698 (20.47)	29 (16–44)	200,312 (20.74)	32 (17–47)	190,582 (19.73)	33 (18–48)	190,335 (19.71)	34 (18–49)	186,978 (19.36)	34 (18–48)	536,507 (55.54)	429,398 (44.46)
CDF -> morbidity	624,823	41 (25–55)	130,538 (20.89)	37 (23–51)	131,411 (21.03)	40 (24–54)	124,983 (20)	41 (25–55)	121,944 (19.52)	43 (27–56)	115,947 (18.56)	43 (27–56)	312,155 (49.96)	312,668 (50.04)
CDF -> multimorbidity	42,453	51 (34–66)	9129 (21.5)	49 (32–65)	9312 (21.93)	51 (34–67)	8279 (19.5)	51 (34–66)	7936 (18.69)	51 (34–66)	7797 (18.37)	52 (35–66)	23,214 (54.68)	19,239 (45.32)
CDF -> death	5913	65 (47–80)	1474 (24.93)	61 (42–76)	1393 (23.56)	65 (46–80)	1126 (19.04)	66 (47–81)	1060 (17.93)	67 (51–82)	860 (14.54)	68 (50–82)	3765 (63.67)	2148 (36.33)
Morbidity -> multimorbidity	423,403	48 (32–61)	89,355 (21.1)	40 (24–54)	89,906 (21.23)	43 (27–57)	84,750 (20.02)	45 (29–58)	82,656 (19.52)	46 (31–59)	76,736 (18.12)	47 (33–59)	197,225 (46.58)	226,178 (53.42)
Morbidity -> death	4519	70 (54–83)	1099 (24.32)	65 (46–79)	1082 (23.94)	68 (51–81)	872 (19.3)	67 (52–81)	861 (19.05)	69 (54–81)	605 (13.39)	68 (55–82)	2594 (57.4)	1925 (42.6)
Multimorbidity -> death	46,053	75 (64–84)	10,432 (22.65)	67 (56–76)	10,704 (23.24)	69 (59–78)	9101 (19.76)	70 (59–78)	8694 (18.88)	70 (60–79)	7122 (15.46)	70 (60–79)	24,487 (53.17)	21,566 (46.83)

CDF = chronic disease free.

Welsh Index of multiple deprivation 2019 income quintiles.

From the chronic disease-free starting state, 624,823 (64.7%) individuals developed one chronic disease, 42,453 (4.4%) developed two or more diseases, 5913 (0.6%) died before developing any of the 132 diseases, and 292,713 (30.3%) remained in the chronic disease-free state for the follow-up duration (Table 1). Some 20.9% of individuals living in the most deprived areas in Wales developed one chronic condition compared to 18.6% in the least deprived areas; 21.5% of individuals living in the most deprived areas developed two or more chronic conditions compared to 18.4% in the least deprived areas; and 24.9% of individuals living in the most deprived areas died before developing any disease compared to 14.5% in the least deprived areas.

Of the 624,823 who developed one chronic disease, 423,403 (67.8%) of these individuals went on to develop at least one more chronic disease to become multimorbid, 4519 (0.7%) died, and 196,901 (31.5%) remained morbid (one chronic disease) until follow-up end. Similar to the transitions from chronic disease-free, a higher proportion of individuals that transitioned from morbidity to multimorbidity or death lived in the more deprived areas (Table 1). The transition from single morbidity to multimorbidity shows a large difference between males and females, with 53.4% of individuals diagnosed with at least one more disease being female. 46,053 (9.9%) individuals transitioned from multimorbidity to death (Table 1). Like other transitions, a higher proportion of individuals who transitioned from multimorbidity to death lived in the most deprived areas in Wales, UK (Table 1).

**Fig. 1:**
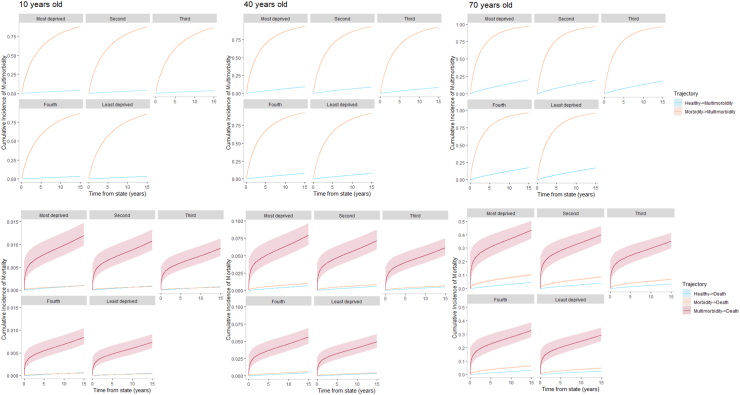
Comparison of the cumulative incidence of multimorbidity and mortality between 10, 40, and 70 year old females by area-level deprivation in Wales.

**Fig. 2:**
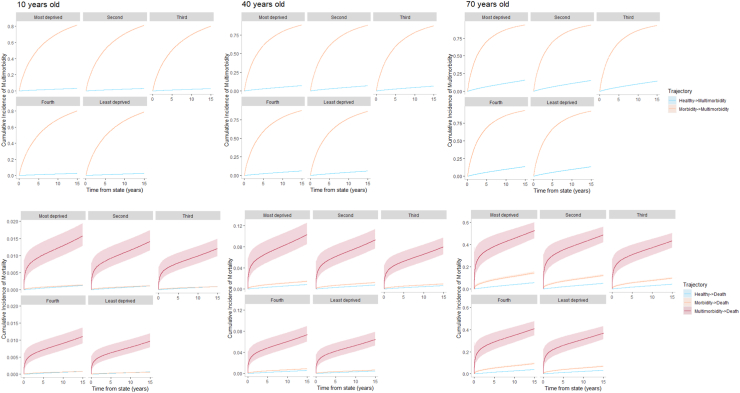
Comparison of the cumulative incidence of multimorbidity and mortality between 10, 40, and 70 year old males by area-level deprivation in Wales.

**Fig. 3:**
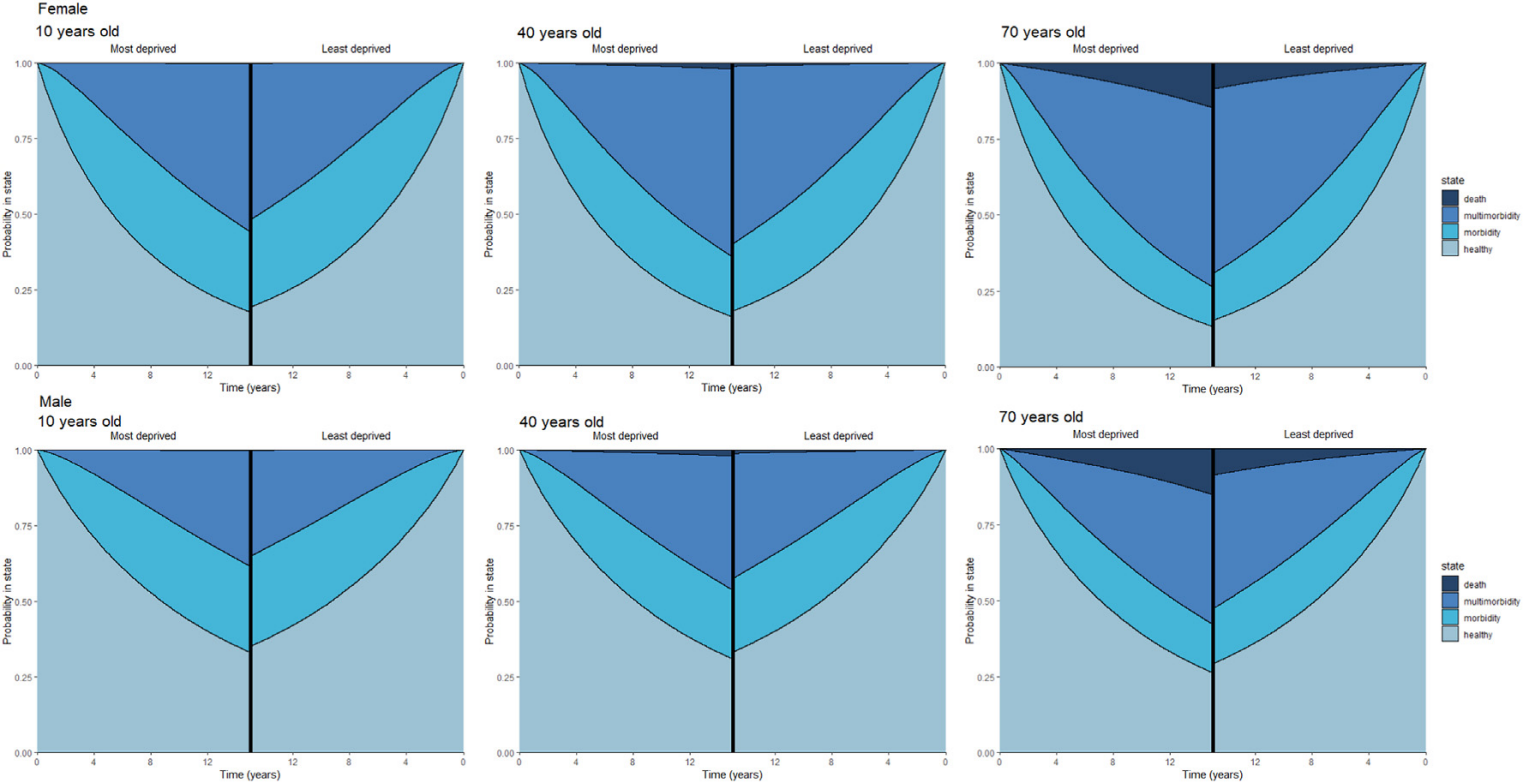
Mirror plot comparing the probability in state over time for 10, 40, and 70 year olds living in one of the most deprived areas of Wales vs 10, 40, and 70 year old living in one of the least deprived areas of Wales by sex.

Overall, across all possible trajectories, ages and sex, those living in the most deprived areas consistently experienced reduced time between states, demonstrating accelerated transitions to chronic morbidity, multimorbidity, and death compared to their equivalent demographic living in the least deprived areas in Wales, UK (Table 2, Supplementary Figure S3). The largest mean difference in time to chronic disease diagnoses of −0.45 years (99% CI: −0.45, −0.44) was observed in younger (10–20 years) males living in the most deprived areas compared to younger males living in the least deprived areas when measuring the trajectory from morbidity to developing multimorbidity. The largest mean difference in time to death of −1.98 years (99% CI: −2.01, −1.95) was observed in 70 year old males living in the most deprived areas compared to 70 year males living in the least deprived areas for the multimorbidity to death trajectory. Taking the youngest, middle age, and oldest individuals as examples, cumulative incidence of multimorbidity, mortality, and probability at each health state were calculated to demonstrate the impact of area level deprivation on accelerating the accrual of disease and mortality over the life-course (Fig. 1, Fig. 2, Fig. 3). Individuals living in the most deprived areas for both males and females spent less time in the initial chronic disease-free state and more time in the morbid, multimorbidity, and death states across the 15 year follow-up. Figures for the other ages and sex can be found in the Supplementary File.

**Table 2:** Restricted Mean Survival Time (RMST) by trajectory and socioeconomic patient group.

Demographic	Trajectory	Most deprived RMST (years)	Second most deprived RMST (years)	Middle deprivation RMST (years)	Second least deprived RMST (years)	Least deprived RMST (years)	Gain (+) or loss (−) in time spent in state for most vs least deprived	99% Confidence intervals of difference		Gain (+) or loss (−) in time spent in state for second most vs least deprived	99% Confidence intervals of difference		Gain (+) or loss (−) in time spent in state for middle vs least deprived	99% Confidence intervals of difference		Gain (+) or loss (−) in time spent in state for second least vs least deprived	99% Confidence intervals of difference	
10 year old female	CDF -> morbidity	6.86	6.87	6.81	6.86	7.09	−0.23	−0.23	−0.23	−0.22	−0.22	−0.22	−0.28	−0.28	−0.28	−0.24	−0.24	−0.24
	CDF -> MM	14.66	14.67	14.69	14.70	14.71	−0.05	−0.05	−0.05	−0.04	−0.04	−0.04	−0.02	−0.02	−0.02	−0.01	−0.01	−0.01
	CDF -> death	14.99	14.99	14.99	14.99	14.99	<−0.01	<−0.01	<−0.01	<−0.01	<−0.01	<−0.01	<−0.01	<−0.01	<−0.01	<−0.01	<−0.01	<−0.01
	Morbidity -> MM	5.32	5.39	5.53	5.55	5.76	−0.44	−0.44	−0.44	−0.37	−0.37	−0.37	−0.22	−0.22	−0.22	−0.20	−0.21	−0.20
	Morbidity -> death	14.98	14.99	14.99	14.99	14.99	−0.01	−0.01	−0.01	<−0.01	<−0.01	<−0.01	<−0.01	<−0.01	<−0.01	<−0.01	<−0.01	<−0.01
	MM -> death	14.86	14.88	14.89	14.90	14.91	−0.05	−0.05	−0.05	−0.04	−0.04	−0.04	−0.02	−0.02	−0.02	−0.01	−0.01	−0.01
10 year old male	CDF -> morbidity	8.89	8.90	8.84	8.89	9.10	−0.21	−0.21	−0.21	−0.20	−0.21	−0.20	−0.26	−0.26	−0.26	−0.22	−0.22	−0.22
	CDF -> MM	14.73	14.74	14.76	14.77	14.77	−0.04	−0.04	−0.04	−0.03	−0.03	−0.03	−0.02	−0.02	−0.02	<−0.01	<−0.01	<−0.01
	CDF -> death	14.98	14.99	14.99	14.99	14.99	<−0.01	<−0.01	<−0.01	<−0.01	<−0.01	<−0.01	<−0.01	<−0.01	<−0.01	<−0.01	<−0.01	<−0.01
	Morbidity -> MM	6.39	6.46	6.61	6.63	6.84	−0.45	−0.45	−0.45	−0.38	−0.38	−0.38	−0.23	−0.23	−0.22	−0.21	−0.21	−0.21
	Morbidity -> death	14.98	14.98	14.99	14.99	14.99	−0.01	−0.01	−0.01	−0.01	−0.01	−0.01	<−0.01	<−0.01	<−0.01	<−0.01	<−0.01	<−0.01
	MM -> death	14.82	14.84	14.86	14.87	14.89	−0.07	−0.07	−0.06	−0.05	−0.05	−0.05	−0.03	−0.03	−0.02	−0.02	−0.02	−0.01
20 year old female	CDF -> morbidity	6.84	6.84	6.78	6.83	7.07	−0.23	−0.23	−0.23	−0.22	−0.22	−0.22	−0.28	−0.28	−0.28	−0.24	−0.24	−0.24
	CDF -> MM	14.55	14.57	14.59	14.61	14.62	−0.07	−0.07	−0.07	−0.06	−0.06	−0.06	−0.03	−0.03	−0.03	−0.01	−0.01	−0.01
	CDF -> death	14.98	14.98	14.98	14.98	14.99	−0.01	−0.01	−0.01	<−0.01	<−0.01	<−0.01	<−0.01	<−0.01	<−0.01	<−0.01	<−0.01	<−0.01
	Morbidity -> MM	4.94	5.01	5.15	5.17	5.37	−0.43	−0.43	−0.43	−0.36	−0.36	−0.36	−0.22	−0.22	−0.22	−0.20	−0.20	−0.20
	Morbidity -> death	14.97	14.98	14.98	14.98	14.98	−0.01	−0.01	−0.01	−0.01	−0.01	−0.01	<−0.01	<−0.01	<−0.01	<−0.01	<−0.01	<−0.01
	MM -> death	14.75	14.77	14.81	14.82	14.84	−0.09	−0.10	−0.09	−0.07	−0.07	−0.07	−0.04	−0.04	−0.04	−0.02	−0.02	−0.02
20 year old male	CDF -> morbidity	8.87	8.88	8.82	8.86	9.08	−0.21	−0.21	−0.21	−0.21	−0.21	−0.20	−0.26	−0.26	−0.26	−0.22	−0.22	−0.22
	CDF -> MM	14.65	14.66	14.68	14.70	14.71	−0.05	−0.05	−0.05	−0.04	−0.04	−0.04	−0.02	−0.02	−0.02	−0.01	−0.01	−0.01
	CDF -> death	14.98	14.98	14.98	14.98	14.98	−0.01	−0.01	−0.01	−0.01	−0.01	−0.01	<−0.01	<−0.01	<−0.01	<−0.01	<−0.01	<−0.01
	Morbidity -> MM	6.00	6.07	6.22	6.24	6.44	−0.45	−0.45	−0.44	−0.38	−0.38	−0.38	−0.23	−0.23	−0.22	−0.21	−0.21	−0.21
	Morbidity -> death	14.96	14.97	14.97	14.98	14.98	−0.02	−0.02	−0.02	−0.01	−0.01	−0.01	−0.01	−0.01	−0.01	<−0.01	<−0.01	<−0.01
	MM -> death	14.67	14.71	14.75	14.77	14.80	−0.12	−0.13	−0.12	−0.09	−0.09	−0.09	−0.05	−0.05	−0.05	−0.03	−0.03	−0.03
30 year old female	CDF -> morbidity	6.81	6.82	6.76	6.81	7.04	−0.23	−0.23	−0.23	−0.22	−0.22	−0.22	−0.28	−0.28	−0.28	−0.24	−0.24	−0.24
	CDF -> MM	14.42	14.43	14.47	14.50	14.51	−0.09	−0.09	−0.09	−0.07	−0.07	−0.07	−0.04	−0.04	−0.04	−0.01	−0.01	−0.01
	CDF -> death	14.97	14.97	14.97	14.97	14.98	−0.01	−0.01	−0.01	−0.01	−0.01	−0.01	−0.01	−0.01	−0.01	<−0.01	<−0.01	<−0.01
	Morbidity -> MM	4.57	4.63	4.77	4.79	4.99	−0.42	−0.42	−0.42	−0.35	−0.36	−0.35	−0.21	−0.22	−0.21	−0.20	−0.20	−0.19
	Morbidity -> death	14.95	14.96	14.96	14.97	14.97	−0.02	−0.02	−0.02	−0.02	−0.02	−0.02	−0.01	−0.01	−0.01	−0.01	−0.01	−0.01
	MM -> death	14.53	14.58	14.64	14.67	14.71	−0.18	−0.18	−0.17	−0.13	−0.13	−0.13	−0.07	−0.07	−0.07	−0.04	−0.04	−0.04
30 year old male	CDF -> morbidity	8.85	8.85	8.80	8.84	9.06	−0.21	−0.21	−0.21	−0.21	−0.21	−0.20	−0.26	−0.26	−0.26	−0.22	−0.22	−0.22
	CDF -> MM	14.55	14.56	14.59	14.61	14.62	−0.07	−0.07	−0.07	−0.06	−0.06	−0.06	−0.03	−0.03	−0.03	−0.01	−0.01	−0.01
	CDF -> death	14.96	14.96	14.97	14.97	14.97	−0.02	−0.02	−0.02	−0.01	−0.01	−0.01	−0.01	−0.01	−0.01	<−0.01	<−0.01	<−0.01
	Morbidity -> MM	5.60	5.67	5.82	5.84	6.05	−0.44	−0.44	−0.44	−0.37	−0.38	−0.37	−0.22	−0.23	−0.22	−0.21	−0.21	−0.20
	Morbidity -> death	14.93	14.94	14.95	14.95	14.96	−0.04	−0.04	−0.04	−0.02	−0.02	−0.02	−0.01	−0.01	−0.01	−0.01	−0.01	−0.01
	MM -> death	14.39	14.45	14.53	14.57	14.62	−0.23	−0.23	−0.23	−0.17	−0.17	−0.17	−0.09	−0.09	−0.09	−0.05	−0.06	−0.05
40 year old female	CDF -> morbidity	6.79	6.79	6.74	6.78	7.02	−0.23	−0.23	−0.23	−0.22	−0.22	−0.22	−0.28	−0.28	−0.28	−0.24	−0.24	−0.24
	CDF -> MM	14.24	14.26	14.31	14.34	14.35	−0.11	−0.12	−0.11	−0.09	−0.10	−0.09	−0.05	−0.05	−0.05	−0.01	−0.01	−0.01
	CDF -> death	14.94	14.95	14.95	14.96	14.96	−0.02	−0.02	−0.02	−0.02	−0.02	−0.02	−0.01	−0.01	−0.01	−0.01	−0.01	−0.01
	Morbidity -> MM	4.21	4.27	4.41	4.42	4.61	−0.40	−0.41	−0.40	−0.34	−0.34	−0.34	−0.21	−0.21	−0.21	−0.19	−0.19	−0.19
	Morbidity -> death	14.90	14.91	14.93	14.93	14.95	−0.05	−0.05	−0.05	−0.04	−0.04	−0.04	−0.02	−0.02	−0.02	−0.02	−0.02	−0.02
	MM -> death	14.13	14.22	14.33	14.38	14.46	−0.33	−0.33	−0.32	−0.24	−0.25	−0.24	−0.13	−0.13	−0.12	−0.08	−0.08	−0.07
40 year old male	CDF -> morbidity	8.82	8.83	8.78	8.82	9.04	−0.21	−0.21	−0.21	−0.21	−0.21	−0.21	−0.26	−0.26	−0.26	−0.22	−0.22	−0.22
	CDF -> MM	14.41	14.42	14.46	14.49	14.50	−0.09	−0.09	−0.09	−0.07	−0.07	−0.07	−0.04	−0.04	−0.04	−0.01	−0.01	−0.01
	CDF -> death	14.93	14.93	14.94	14.95	14.96	−0.03	−0.03	−0.03	−0.02	−0.02	−0.02	−0.01	−0.01	−0.01	−0.01	−0.01	−0.01
	Morbidity -> MM	5.22	5.29	5.43	5.45	5.65	−0.44	−0.44	−0.43	−0.37	−0.37	−0.37	−0.22	−0.22	−0.22	−0.20	−0.20	−0.20
	Morbidity -> death	14.85	14.88	14.90	14.91	14.93	−0.08	−0.08	−0.08	−0.05	−0.05	−0.05	−0.03	−0.03	−0.03	−0.02	−0.02	−0.02
	MM -> death	13.87	13.98	14.12	14.19	14.29	−0.42	−0.43	−0.42	−0.31	−0.32	−0.31	−0.17	−0.17	−0.16	−0.10	−0.10	−0.09
50 year old female	CDF -> morbidity	6.76	6.77	6.71	6.76	6.99	−0.23	−0.23	−0.23	−0.22	−0.22	−0.22	−0.28	−0.28	−0.28	−0.24	−0.24	−0.24
	CDF -> MM	14.01	14.03	14.10	14.14	14.16	−0.15	−0.15	−0.15	−0.12	−0.12	−0.12	−0.06	−0.06	−0.06	−0.02	−0.02	−0.02
	CDF -> death	14.89	14.90	14.92	14.93	14.94	−0.04	−0.04	−0.04	−0.03	−0.03	−0.03	−0.02	−0.02	−0.02	−0.01	−0.01	−0.01
	Morbidity -> MM	3.87	3.93	4.06	4.07	4.25	−0.39	−0.39	−0.39	−0.33	−0.33	−0.33	−0.20	−0.20	−0.20	−0.18	−0.18	−0.18
	Morbidity -> death	14.78	14.81	14.85	14.86	14.90	−0.12	−0.12	−0.12	−0.08	−0.08	−0.08	−0.04	−0.04	−0.04	−0.04	−0.04	−0.04
	MM -> death	13.40	13.55	13.76	13.85	13.99	−0.59	−0.60	−0.59	−0.44	−0.45	−0.43	−0.24	−0.24	−0.23	−0.14	−0.15	−0.13
50 year old male	CDF -> morbidity	8.80	8.81	8.75	8.80	9.01	−0.21	−0.22	−0.21	−0.21	−0.21	−0.21	−0.26	−0.26	−0.26	−0.22	−0.22	−0.22
	CDF -> MM	14.23	14.25	14.30	14.33	14.34	−0.12	−0.12	−0.12	−0.10	−0.10	−0.10	−0.05	−0.05	−0.05	−0.01	−0.01	−0.01
	CDF -> death	14.87	14.88	14.90	14.91	14.92	−0.06	−0.06	−0.05	−0.04	−0.04	−0.04	−0.02	−0.02	−0.02	−0.02	−0.02	−0.02
	Morbidity -> MM	4.84	4.91	5.05	5.07	5.27	−0.43	−0.43	−0.42	−0.36	−0.36	−0.36	−0.22	−0.22	−0.22	−0.20	−0.20	−0.20
	Morbidity -> death	14.68	14.74	14.79	14.80	14.85	−0.17	−0.17	−0.17	−0.12	−0.12	−0.12	−0.06	−0.06	−0.06	−0.05	−0.05	−0.05
	MM -> death	12.93	13.13	13.39	13.51	13.69	−0.76	−0.77	−0.75	−0.56	−0.57	−0.55	−0.30	−0.31	−0.29	−0.18	−0.19	−0.17
60 year old female	CDF -> morbidity	6.74	6.75	6.69	6.73	6.97	−0.23	−0.23	−0.23	−0.22	−0.22	−0.22	−0.28	−0.28	−0.28	−0.24	−0.24	−0.24
	CDF -> MM	13.71	13.74	13.82	13.88	13.90	−0.19	−0.19	−0.19	−0.16	−0.16	−0.16	−0.08	−0.08	−0.08	−0.02	−0.02	−0.02
	CDF -> death	14.80	14.83	14.85	14.86	14.89	−0.08	−0.08	−0.08	−0.06	−0.06	−0.06	−0.04	−0.04	−0.04	−0.02	−0.02	−0.02
	Morbidity -> MM	3.54	3.60	3.72	3.74	3.91	−0.37	−0.37	−0.37	−0.31	−0.32	−0.31	−0.19	−0.19	−0.19	−0.17	−0.18	−0.17
	Morbidity -> death	14.52	14.60	14.68	14.70	14.78	−0.26	−0.26	−0.26	−0.18	−0.18	−0.18	−0.10	−0.10	−0.10	−0.08	−0.08	−0.08
	MM -> death	12.11	12.38	12.73	12.90	13.15	−1.04	−1.05	−1.02	−0.77	−0.78	−0.75	−0.42	−0.43	−0.40	−0.25	−0.26	−0.23
60 year old male	CDF -> morbidity	8.78	8.78	8.73	8.77	8.99	−0.21	−0.22	−0.21	−0.21	−0.21	−0.21	−0.26	−0.26	−0.26	−0.22	−0.22	−0.22
	CDF -> MM	13.99	14.02	14.08	14.13	14.14	−0.15	−0.15	−0.15	−0.12	−0.13	−0.12	−0.06	−0.06	−0.06	−0.02	−0.02	−0.02
	CDF -> death	14.75	14.78	14.81	14.83	14.86	−0.10	−0.10	−0.10	−0.08	−0.08	−0.08	−0.04	−0.04	−0.04	−0.03	−0.03	−0.03
	Morbidity -> MM	4.47	4.54	4.67	4.69	4.89	−0.41	−0.42	−0.41	−0.35	−0.35	−0.35	−0.21	−0.21	−0.21	−0.19	−0.20	−0.19
	Morbidity -> death	14.32	14.43	14.55	14.57	14.69	−0.37	−0.37	−0.36	−0.26	−0.26	−0.25	−0.14	−0.14	−0.14	−0.11	−0.11	−0.11
	MM -> death	11.33	11.66	12.10	12.31	12.62	−1.29	−1.31	−1.27	−0.96	−0.98	−0.94	−0.52	−0.54	−0.50	−0.31	−0.33	−0.29
70 year old female	CDF -> morbidity	6.71	6.72	6.66	6.71	6.95	−0.23	−0.23	−0.23	−0.22	−0.23	−0.22	−0.28	−0.28	−0.28	−0.24	−0.24	−0.24
	CDF -> MM	13.33	13.37	13.48	13.55	13.58	−0.24	−0.24	−0.24	−0.20	−0.20	−0.20	−0.10	−0.10	−0.10	−0.03	−0.03	−0.03
	CDF -> death	14.64	14.68	14.72	14.75	14.79	−0.15	−0.16	−0.15	−0.11	−0.12	−0.11	−0.07	−0.07	−0.07	−0.04	−0.04	−0.04
	Morbidity -> MM	3.23	3.28	3.40	3.41	3.58	−0.35	−0.35	−0.35	−0.30	−0.30	−0.30	−0.18	−0.18	−0.18	−0.17	−0.17	−0.16
	Morbidity -> death	13.98	14.15	14.32	14.36	14.53	−0.55	−0.55	−0.55	−0.38	−0.39	−0.38	−0.21	−0.21	−0.21	−0.17	−0.17	−0.17
	MM -> death	10.02	10.44	11.01	11.28	11.69	−1.67	−1.70	−1.65	−1.25	−1.28	−1.23	−0.69	−0.71	−0.66	−0.41	−0.43	−0.39
70 year old male	CDF -> morbidity	8.75	8.76	8.71	8.75	8.97	−0.22	−0.22	−0.21	−0.21	−0.21	−0.21	−0.26	−0.26	−0.26	−0.22	−0.22	−0.22
	CDF -> MM	13.69	13.72	13.81	13.86	13.88	−0.19	−0.19	−0.19	−0.16	−0.16	−0.16	−0.08	−0.08	−0.08	−0.02	−0.02	−0.02
	CDF -> death	14.54	14.59	14.65	14.68	14.74	−0.19	−0.20	−0.19	−0.14	−0.14	−0.14	−0.08	−0.08	−0.08	−0.05	−0.05	−0.05
	Morbidity -> MM	4.12	4.18	4.31	4.33	4.52	−0.40	−0.40	−0.40	−0.34	−0.34	−0.34	−0.20	−0.21	−0.20	−0.19	−0.19	−0.19
	Morbidity -> death	13.56	13.79	14.03	14.09	14.33	−0.77	−0.78	−0.77	−0.54	−0.54	−0.54	−0.29	−0.30	−0.29	−0.24	−0.24	−0.24
	MM -> death	8.84	9.33	10.00	10.33	10.82	−1.98	−2.01	−1.95	−1.49	−1.52	−1.46	−0.82	−0.85	−0.80	−0.49	−0.52	−0.47

Max RMST = 15 years.

MM = multimorbidity.

CDF = chronic disease-free.

Across all ages and sex, individuals living in the most deprived areas lost approximately a fifth of a year from developing a chronic disease (Table 2, Supplementary Figure S3). On average, 10 year old females living in deprived areas spent 6.86 years disease-free compared to 7.09 years disease-free years to their demographic equivalent living in a least deprived area. Similar trends can also be seen in 10 year old males. However, compared to females, 10 year old males spend more time in the chronic disease-free state, with a RMST of 8.89 and 9.10 for the most and least deprived respectively. This observation and comparison between males and females of the same age can also be seen across all age groups. Increasing age is also associated with accelerated rates of transition to morbidity.

Development of multimorbidity from both a chronic disease-free state and single morbidity state resulted in a reduction in RMST, subsequent acceleration to multimorbidity, of −0.04 (99% CI: −0.04, −0.04) to −0.45 (99% CI: −0.45, −0.44) years across age and sex in individuals living in the most deprived areas compared to their demographic equivalent living in a least deprived area (Table 2, Supplementary Figure S3). Taking 40 year old females as an example, those living in the most deprived areas spend 6.79 years chronic disease-free compared to 7.02 years for those living in the least deprived, a reduction of −0.23 years (99% CI: −0.23, −0.23). Once they had already accrued a chronic disease, 40 year old females living in more deprived areas spent on average 4.21 years before developing further chronic diseases compared to 4.61 years for females living in the least deprived areas, a reduction in time of −0.40 years (99% CI: −0.41, −0.40). This difference is also seen in 40 year old males, however, progression to multimorbidity occurs later compared to females.

Across the three possible trajectories to death (Supplementary Figure S2), individuals living in the most deprived areas experience accelerated transitions for both male and females, and across all ages when compared to individuals living in the least deprived areas (Table 2, Supplementary Figure S3). The RMST ranges from a small difference of <−0.01 (99% CI: <−0.01, <−0.01) years in 10 year olds to −1.98 (99% CI: −2.01, −1.95) years for 70 year old males living in more deprived areas compared to their demographic equivalent living in a least deprived area. Males consistently experience accelerated transitions to death compared to females. Regardless of sex, multimorbidity accelerated the transition to death for all ages which is further accelerated for individuals living in the most deprived areas.

## Discussion

This study was designed to examine the differences in trajectories of single and 2+ chronic disease accrual to death for the population of Wales, UK and to explore how this differed by area-based deprivation status and sex. The study incorporated 12.7 million person-years of follow-up on 965,905 people, with an average individual follow up of 13.2 years.

Overall, across all the possible trajectories, regardless of age and sex, people living in the most deprived areas consistently spent less time in the chronic disease-free state as well as between single morbidity to multimorbidity, and in any state before dying compared to their demographic equivalent living in the least deprived areas. Differences in RMST between people living in the most and least deprived areas ranged from a small difference of less than −0.01 in the younger chronic disease-free individuals when measuring time to death to −1.98 in older individuals for the trajectory between multimorbidity to death (Table 2). Results show accelerated rates of transition from being chronic disease-free to developing morbidity, multimorbidity, and death in individuals living in more deprived areas across both sexes and for all age groups, demonstrating faster accrual of chronic disease and death.

The results also show that males spend more time chronic disease-free compared to females of the same age and area-level deprivation but experience accelerated time to death. Whilst the time spent chronic disease-free can be perceived to be a positive outcome, the reduction in time to death suggests that this finding could be masking delays in diagnoses, resulting in unmanaged conditions contributing to poorer health and health outcomes as well as highlight differences in disease developments between sexes.

Previous multimorbidity trajectory studies did not use a clearance period to identify the disease-free state. The inclusion of a clearance period reduces bias when identifying true incident conditions compared to prevalent cases. There is a trade-off between the clearance duration and follow up periods available. After exploration, we used a 5-year clearance and conducted sensitivity analyses for 1-year and 10-year clearances, as well as measuring deprivation at the start of the study (Supplementary Tables S3–S5). The sensitivity analyses where the clearance period was reduced to 1 year or increased to 10 years resulted in differences in the absolute values of RMST due to varying the total follow-up time compared to the main analysis, as expected. However, whilst the RMST differed, the relative difference in RMST between area-level deprivation and by age and sex were little changed. Similarly, the sensitivity analysis where deprivation was measured at the start of the study produced the comparable results as the main analysis which also supports the main analyses of the study (Table 2, Supplementary Table S3).

Among the strengths of this study was the ability to link multiple demographic, longitudinal primary and secondary healthcare, and mortality data for the population of Wales through the SAIL Databank.^20^^,^^21^ Having access to the underlying individual-level data allowed inclusion of disease diagnosis across healthcare settings for 132 chronic diseases and allowed the inclusion of covariates such as age as a time-varying covariate. Previous studies have had to rely on a set age at the study start or end date, or included health data from primary care only, included a limited number of conditions or disease specific and only focussed on outcomes in adults.^4^^,^^12^^,^^32^ Additionally, with access to 20 years of EHRs for the population of Wales, we were able to include a clearance period in our study to follow all individuals from an equal chronic disease-free starting state and capturing first disease occurrence, subsequent disease diagnoses and any deaths recorded from 1st January 2005.

Although our study provides important findings on the different trajectories of health outcomes between the most and least deprived individuals in Wales, several limitations need to be considered. In this study, we focussed on measuring the difference between individuals from affluent or deprived areas in developing chronic diseases over time who were disease-free up to the starting point of the study. With all individuals having to survive to 1st January 2005 and the starting point for analysis being the same for all age-sex categories (to reduce bias of prior health conditions at baseline influencing the analysis), this selection criteria for our study will have reduced the numbers included, particularly in the older age groups. It is important to realise that this study was not designed to measure socioeconomic differences in chronic disease prevalence as there is already an extensive literature on that.

Whilst 132 chronic conditions have been included in this analysis, this is not a comprehensive list of all chronic conditions. The CALIBER list of phenotypes was the most extensive list available to us at study design.^25^ Nevertheless, it is unlikely that the patterns of health disparities we identified would be changed by the inclusion of other conditions. Diagnoses prior to 2000 were not included as this timeframe falls outside the WMC membership follow-up period and historic EHR may not be complete, therefore, some historical diagnoses cannot be accounted for. It was not possible to account for any delays from disease development to recording of diagnosis due to patient specific healthcare interactions. Similarly, there are likely to be missed diagnoses. In the analysis of computerised records it is not possible to account for self-medication of conditions instead of seeking medical help resulting in missed recordings of diagnoses. It is possible that there are different rates of use of ‘over the counter’ treatments that will vary by the ability to pay for treatment. However, this is less of an issue in Wales compared to the rest of the UK as all prescriptions are free.

A further potential limitation of this study is we could not account for disease severity or resolution. The 132 conditions included from the CALIBER resource were chosen with this in mind. Most medical ontology terms do not capture severity of disease well. Additionally, as we could not accounting for disease severity, it is not possible to measure burdensome as each disease included without weighting and so does not reflect the impact of conditions on individual’s health and wellbeing. Some conditions will be more serious or burdensome than others in general but are not captured by disease classifications used in hospitals or GPs.

We did not include ethnicity in the models due to poor coding in GP records, however, we plan to explore ethnicity in future work once the ONS Census 2021 data becomes available to improve ethnicity recording particularly for ethnic minority groups where sample sizes are small due to the predominantly white population in Wales, UK.

Lastly, this study utilised relative area-level deprivation quintiles at the study end which will underestimate the effect of individual-level deprivation as shown in a Danish birth cohort.^33^ However, previous research investigating the relationship between health and area and individual-level measures of deprivation in Wales reported that area-level differences persist after adjustment for factors such as individual measures of social class, employment status, family income, household tenure and council tax band.^34^

These results have important policy implications. In this study, 69% developed at least one new chronic disease demonstrating substantial population-wide accrual of morbidity over the life-course. Taking one of the demographic groups in this study as an example, 10 year olds expect to be diagnosed with their first chronic disease on average between 6.86–7.09 years for females living in the most and least deprived areas, and 8.89–9.10 years for males living in the most and least deprived areas. The differences between the most and least deprived individuals provide guidance on ages of preventative intervention and highlight an important inequity in our society that should be addressed through adequate policy responses for prevention and treatment, and provision of services.^35^

This study contributes to the evidence base for demonstrating both reduced time spent free of chronic diseases and accelerated time to death in deprived individuals living with chronic diseases.^36^^,^^37^ To our knowledge, this study is the first to examine the accrual of multiple chronic diseases and associated mortality by socioeconomic status in a population-wide, long-term study having included a clearance period and adjusted for competing risks.

The results provide quantifiable evidence in terms of restricted mean survival times (time spent in states) with respect to health inequity across all ages and sexes, which will be used to support better public and policy understanding of the implications of social determinants of health. As previously mentioned, further work will include the incorporation of ethnicity into future models to explore the difference in disease pathways and health equity by ethnic groups and individual-level metrics of deprivation.

In conclusion, this study has provided quantifiable evidence of the disparity in the speed of accruing chronic diseases and early mortality by area deprivation status across all ages, demonstrating that individuals living in more deprived areas experience accelerated transitions to chronic disease(s) diagnosis and mortality. Outputs from this study can be used to inform social policy and clinical practice by highlighting the scale of the health inequity and help identify marginalised groups of individuals for intervention.

## Contributors

JL, RAL, and RKO conceived and designed the study. JL and RKO had full access to all data used in this study. Due to data permission restrictions, not all authors were able to access the underlying data used in the study. JL checked and verified the data used in the analysis, and conducted the analysis in consultation with RKO. JL wrote the original draft. JL, AA, KRA, AAL, TBD, JC, SD, RF, CPG, JG, LJG, BG, MH, FJ, AJ, CM, CMC, NP, DOR, JR, RAL, and RKO reviewed, edited, and approved the final manuscript. All authors were responsible for submitting the article for publication.

## Data sharing statement

This study makes use of anonymised, individual-level data held in the SAIL Databank, a Trusted Research Environment, at Swansea University, Swansea, UK. Due to the nature and level of the data, data are not publicly available. All proposals to use SAIL data are subject to review by the independent Information Governance Review Panel (IGRP). The IGRP gives careful consideration to each project proposal to ensure proper and appropriate use of SAIL data. If a project is approved, access to the requested data is gained through a privacy-protecting safe haven and remote access system referred to as the SAIL Gateway. SAIL has established an application process to be followed by anyone who would like to access data via SAIL at: https://www.saildatabank.com/application-process/.

## Declaration of interests

RKO is a member of the National Institute for Health and Care Excellence (NICE) Technology Appraisal Committee, member of the NICE Decision Support Unit (DSU), and associate member of the NICE Technical Support Unit (TSU). She has served as a paid consultant providing unrelated methodological advice to AstraZeneca, Cogentia Healthcare Ltd., Daiichi Sankyo, NICE, Norwegian Institute of Public Health, Roche, and Vifor Pharma. She reports teaching fees from the Association of British Pharmaceutical Industry (ABPI) and the University of Bristol.

KRA is a member of the National Institute for Health and Care Excellence (NICE) Diagnostics Advisory Committee, the NICE Decision and Technical Support Units, and is a National Institute for Health Research (NIHR) Senior Investigator Emeritus [NF-SI-0512-10159]. He has served as a paid consultant, providing unrelated methodological and strategic advice, to the pharmaceutical and life sciences industry generally, as well as to DHSC/NICE, and has received unrelated research funding from Association of the British Pharmaceutical Industry (ABPI), European Federation of Pharmaceutical Industries & Associations (EFPIA), Pfizer, Sanofi and Swiss Precision Diagnostics/Clearblue. He has also received course fees from ABPI and is a Partner and Director of Visible Analytics Limited, a health technology assessment consultancy company.

CMC has received previous funding from Medical Research Council, National Institute for Health and Care Research, Wellcome Trust, and Chief Scientists Office.

JR has received previous funding from Medical Research Council, and NHS England for an internship programmed collaboration.

CPG has received funding from Abbott Diabetes, National Institute for Health and Care Research, BMS, and BHF. He has served as paid consultant for Amgen, AINexus, AstraZeneica, Bayer, BMS, Cardiomatics, Chessi, Diachii Sankyo, iRhythm, and Organon. He has also received payment from AstraZeneca, Bayer, Novartis, WomdrMedical, and Zydos, and received materials from Echonous and Kosmos.

All other authors report no conflicts of interest.

## References

[1] Hosseinpoor A.R., Bergen N., Schlotheuber A. (2015). Promoting health equity: WHO health inequality monitoring at global and national levels. Glob Health Action.

[2] Kivimäki M., Batty G.D., Pentti J. (2020). Association between socioeconomic status and the development of mental and physical health conditions in adulthood: a multi-cohort study. Lancet Public Health.

[3] McMaughan D.J., Oloruntoba O., Smith M.L. (2020). Socioeconomic status and access to healthcare: interrelated drivers for healthy aging. Front Public Health.

[4] Dugravot A., Fayosse A., Dumurgier J. (2020). Social inequalities in multimorbidity, frailty, disability, and transitions to mortality: a 24-year follow-up of the Whitehall II cohort study. Lancet Public Health.

[5] Cezard G., Sullivan F., Keenan K. (2022). Understanding multimorbidity trajectories in Scotland using sequence analysis. Sci Rep.

[6] The Academy of Medical Sciences (2018). https://acmedsci.ac.uk/filedownload/82222577.

[7] Wallace E., Salisbury C., Guthrie B., Lewis C., Fahey T., Smith S.M. (2015). Managing patients with multimorbidity in primary care. BMJ.

[8] Menotti A., Mulder I., Nissinen A., Giampaoli S., Feskens E.J.M., Kromhout D. (2001). Prevalence of morbidity and multimorbidity in elderly male populations and their impact on 10-year all-cause mortality. J Clin Epidemiol.

[9] Aggarwal P., Woolford S.J., Patel H.P. (2020). Multi-morbidity and polypharmacy in older people: challenges and opportunities for clinical practice. Geriatrics.

[10] West J.S., Lynch S.M. (2020). Demographic and socioeconomic disparities in life expectancy with hearing impairment in the United States. J Gerontol Ser B.

[11] Singh-Manoux A., Fayosse A., Sabia S. (2018). Clinical, socioeconomic, and behavioural factors at age 50 years and risk of cardiometabolic multimorbidity and mortality: a cohort study. PLoS Med.

[12] Höhn A., McGurnaghan S.J., Caparrotta T.M. (2022). Large socioeconomic gap in period life expectancy and life years spent with complications of diabetes in the Scottish population with type 1 diabetes, 2013–2018. PLoS One.

[13] Makovski T.T., Schmitz S., Zeegers M.P., Stranges S., van den Akker M. (2019). Multimorbidity and quality of life: systematic literature review and meta-analysis. Ageing Res Rev.

[14] Chua Y.P., Xie Y., Lee P.S., Lee E.S. (2021). Definitions and prevalence of multimorbidity in large database studies: a scoping review. Int J Environ Res Public Health.

[15] Plana-Ripoll O., Dreier J.W., Momen N.C. (2022). Analysis of mortality metrics associated with a comprehensive range of disorders in Denmark, 2000 to 2018: a population-based cohort study. PLoS Med.

[16] Neumann J.T., Thao L.T., Callander E. (2022). A multistate model of health transitions in older people: a secondary analysis of ASPREE clinical trial data. Lancet Healthy Longev.

[17] Meyer A.C., Ebeling M., Drefahl S. (2022). The impact of hip fracture on geriatric care and mortality among older Swedes: mapping care trajectories and their determinants. Am J Epidemiol.

[18] Ansah J.P., Chiu C.-T. (2023). Projecting the chronic disease burden among the adult population in the United States using a multi-state population model. Front Public Health.

[19] Lyons J., Akbari A., Agrawal U. (2021). Protocol for the development of the Wales multimorbidity e-cohort (WMC): data sources and methods to construct a population-based research platform to investigate multimorbidity. BMJ Open.

[20] Lyons R.A., Jones K.H., John G. (2009). The SAIL databank: linking multiple health and social care datasets. BMC Med Inform Decis Mak.

[21] Ford D.V., Jones K.H., Verplancke J.-P. (2009). The SAIL databank: building a national architecture for e-health research and evaluation. BMC Health Serv Res.

[22] Andersen P.K., Keiding N. (2002). Multi-state models for event history analysis. Stat Methods Med Res.

[23] Owen R., Lyons J., Akbari A. (2022).

[24] Welsh Government Welsh index multiple deprivation index. https://gov.wales/welsh-index-multiple-deprivation-indexguidance.

[25] Kuan V., Denaxas S., Gonzalez-Izquierdo A. (2019). A chronological map of 308 physical and mental health conditions from 4 million individuals in the English National Health Service. Lancet Digit Health.

[26] Andersen P.K., Geskus R.B., de Witte T., Putter H. (2012). Competing risks in epidemiology: possibilities and pitfalls. Int J Epidemiol.

[27] Royston P., Parmar M.K. (2013). Restricted mean survival time: an alternative to the hazard ratio for the design and analysis of randomized trials with a time-to-event outcome. BMC Med Res Methodol.

[28] McCaw Z.R., Yin G., Wei L.-J. (2019). Using the restricted mean survival time difference as an alternative to the hazard ratio for analyzing clinical cardiovascular studies. Circulation.

[29] Therneau T.M., Grambsch P.M. (2013).

[30] Cole S.R., Hudgens M.G., Brookhart M.A., Westreich D. (2015). Risk. Am J Epidemiol.

[31] de Wreede L.C., Fiocco M., Putter H. (2011). mstate: an R package for the analysis of competing risks and multi-state models. J Stat Softw.

[32] Bisquera A., Turner E.B., Ledwaba-Chapman L. (2022). Inequalities in developing multimorbidity over time: a population-based cohort study from an urban, multi-ethnic borough in the United Kingdom. Lancet Reg Health Eur.

[33] Hakulinen C., Mok P.L., Horsdal H.T. (2020). Parental income as a marker for socioeconomic position during childhood and later risk of developing a secondary care-diagnosed mental disorder examined across the full diagnostic spectrum: a national cohort study. BMC Med.

[34] Fone D., Dunstan F., Lloyd K., Williams G., Watkins J., Palmer S. (2007). Does social cohesion modify the association between area income deprivation and mental health? A multilevel analysis. Int J Epidemiol.

[35] Barlow P., Mohan G., Nolan A., Lyons S. (2021). Area-level deprivation and geographic factors influencing utilisation of general practitioner services. SSM Popul Health.

[36] Chan M.S., van den Hout A., Pujades-Rodriguez M. (2019). Socio-economic inequalities in life expectancy of older adults with and without multimorbidity: a record linkage study of 1.1 million people in England. Int J Epidemiol.

[37] McLean G., Gunn J., Wyke S. (2014). The influence of socioeconomic deprivation on multimorbidity at different ages: a cross-sectional study. Br J Gen Pract.

