# Supplementary material for: Trajectories in chronic disease accrual and mortality across the lifespan in Wales, UK (2005–2019), by area deprivation profile: linked electronic health records cohort study on 965,905 individuals
Source: Lancet Reg Health Eur. 2023 Jul 17;32:100687. doi: 10.1016/j.lanepe.2023.100687 (PMC10372901; doi:10.1016/j.lanepe.2023.100687)

Supplementary figures and tables

Contents

**Supplementary Table 1**	**Page 2-6**
**Supplementary Figure 1**	**Page 7**
**Supplementary Table 2**	**Page 8**
**Supplementary Figure 2**	**Page 9**
**Supplementary Table 3**	**Page 10-13**
**Supplementary Table 4**	**Page 14-16**
**Supplementary Table 5**	**Page 17-20**
**Supplementary Table 6**	**Page 21-22**
**Supplementary Table 7**	**Page 23-26**
**Supplementary Table 8**	**Page 27-29**
**Supplementary Figure 3**	**Page 30**
**Supplementary Figure 4**	**Page 31**
**Supplementary Figure 5**	**Page 32**
**Supplementary Figure 6**	**Page 33**
**Supplementary Figure 7**	**Page 34**

Supplementary Table 1: Number and percentages of chronic diseases included in the study follow-up.

Disease	Count	Percentage
Dermatitis (atopc/contact/other/unspecified)	156618	16.21
Hypertension	140151	14.51
Primary Malignancy	129387	13.40
Enthesopathies & synovial disorders	124883	12.93
Gastro-oesoph disorder	78013	8.08
Allergic and chronic rhinitis	77455	8.02
Osteoarthritis (excl spine)	74813	7.75
Asthma	62417	6.46
Anaemias	58201	6.03
Obesity	52411	5.43
Hearing loss	51560	5.34
Menorrhagia and polymenorrhoea	46054	4.77
Migraine	43658	4.52
Diabetes	42922	4.44
Abdominal Hernia	37574	3.89
Erectile dysfunction	34406	3.56
Cataract	34062	3.53
Osteoporosis	33495	3.47
Irritable bowel syndrome	32898	3.41
Coronary heart disease	32704	3.39
Hypo or hyperthyroidism	29993	3.11
high cholesterol	27737	2.87
COPD	27644	2.86
Diverticular disease of intestine (acute and chronic)	27528	2.85
Chronic sinusitis	26867	2.78

Postviral fatigue syndrome, neurasthenia and fibromyalgia	26067	2.70
Cholelithiasis	25016	2.59
CKD	23948	2.48
Psoriasis	23796	2.46
Alcohol Problems	22850	2.37
Secondary Malignancy	19880	2.06
Rosacea	19846	2.05
Hyperplasia of prostate	18790	1.95
Dysmenorrhoea	18787	1.95
Spondylosis	18597	1.93
Actinic keratosis	18017	1.87
Urinary Incontinence	17802	1.84
Heart failure	17087	1.77
Other psychoactive substance misuse	16877	1.75
Heart valve disorders	13794	1.43
Female genital prolapse	13463	1.39
Urolithiasis	12721	1.32
Peripheral neuropathies (excluding cranial nerve and carpal tunnel syndromes)	12662	1.31
Dementia	11689	1.21
Peripheral arterial disease	10465	1.08
Glaucoma	9031	0.93
Stroke	8747	0.91
Epilepsy	8495	0.88
Fibromatoses	7509	0.78
Macular degeneration	7394	0.77
Raynaud's syndrome	7354	0.76
Haemangioma, any site	7096	0.73
Polycystic ovarian syndrome	6875	0.71
Sleep apnoea	6565	0.68

Respiratory failure	6511	0.67
Rheumatoid Arthritis	6210	0.64
Female pelvic inflammatory disease	6057	0.63
Obstructive and reflux uropathy	5802	0.60
Endometriosis	5707	0.59
Visual impairment and blindness	5297	0.55
Schizophrenia, schizotypal and delusional disorders	4952	0.51
Infertility	4823	0.50
Fatty Liver	4675	0.48
Neuromuscular dysfunction of bladder	4615	0.48
Polymyalgia Rheumatica	4454	0.46
Spinal stenosis	4257	0.44
Liver fibrosis, sclerosis and cirrhosis	4012	0.42
Ulcerative colitis	3800	0.39
Intellectual disability	3550	0.37
Alcoholic liver disease	3543	0.37
Barrett's oesophagus	3541	0.37
Keratitis	3529	0.37
Scoliosis	3453	0.36
Obsessive-compulsive disorder	3411	0.35
Personality disorders	3328	0.34
Abdominal aortic aneurysm	3287	0.34
Cardiomyopathy	2739	0.28
Parkinson's disease	2627	0.27
Tubulo-interstitial nephritis	2564	0.27
Crohn's disease	2487	0.26
Hyperkinetic disorders	2484	0.26
Coeliac disease	2378	0.25
Bronchiectasis	2369	0.25

Autism and Asperger's syndrome	2344	0.24
Alopecia areata	2295	0.24
Lichen planus	2264	0.23
Collapsed vertebra	2237	0.23
Other interstitial pulmonary diseases with fibrosis	2072	0.21
End stage renal disease	2036	0.21
Endometrial hyperplasia and hypertrophy	1966	0.20
Vitiligo	1936	0.20
Spondylolisthesis	1612	0.17
Hepatic failure	1600	0.17
Meniere disease	1565	0.16
Anorexia and bulimia nervosa	1544	0.16
Oesophageal varices	1443	0.15
Hyperparathyroidism	1429	0.15
Multiple sclerosis	1423	0.15
Portal hypertension	1343	0.14
Pleural plaque	1315	0.14
Anorectal prolapse	1314	0.14
Disorders of autonomic nervous system	1305	0.14
Psoriatic arthropathy	1286	0.13
Sarcoidosis	1165	0.12
Secondary polycythaemia	1044	0.11
Ankylosing spondylitis	996	0.10
Non-acute cystitis	989	0.10
Polycythaemia vera	969	0.10
Giant Cell arteritis	938	0.10
Chronic viral hepatitis	885	0.09
Secondary pulmonary hypertension	840	0.09
Hyposplenism	836	0.09

Multiple myeloma and malignant plasma cell neoplasms	787	0.08
Primary pulmonary hypertension	729	0.08
Angiodysplasia of colon	706	0.07
Lupus erythematosus (local and systemic)	650	0.07
Cerebral Palsy	588	0.06
Spina bifida	586	0.06
Myelodysplastic syndromes	573	0.06
Autoimmune liver disease	542	0.06
Postinfective and reactive arthropathies	527	0.05
Sjogren's disease	483	0.05
Syndrome of inappropriate secretion of antidiuretic hormone	458	0.05
Motor neuron disease	443	0.05
Asbestosis	318	0.03
Down's syndrome	307	0.03
Immunodeficiencies	258	0.03
Myasthenia gravis	228	0.02
Systemic sclerosis	204	0.02
Juvenile arthritis	170	0.02
Cystic Fibrosis	93	0.01
Enteropathic arthropathy	46	0.00

Supplementary Figure 1: Consort diagram of study participant inclusion.

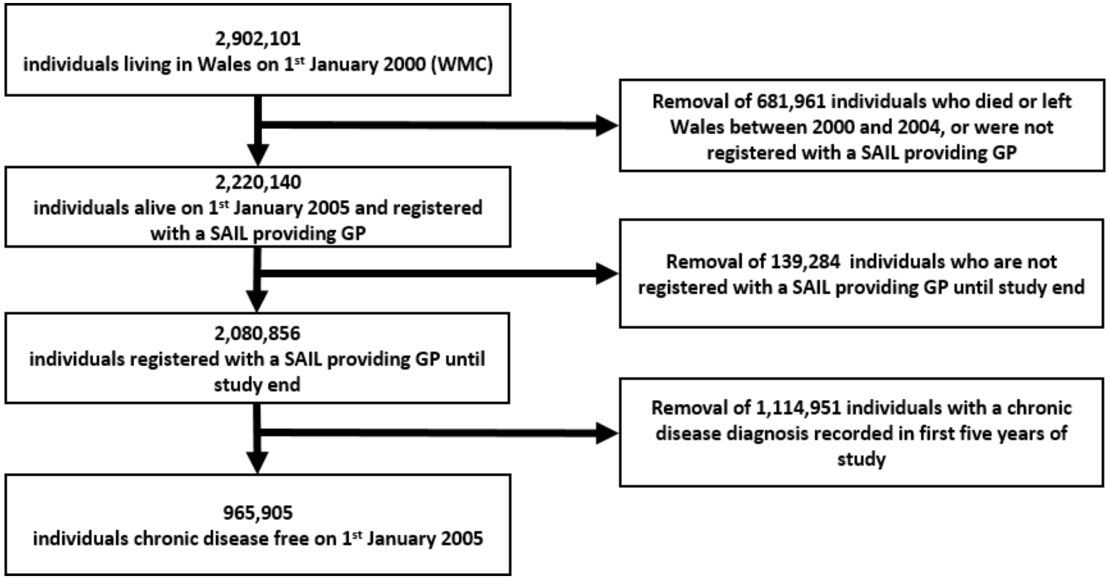

Supplementary Table 2: Number of study participants registered with a SAIL practice by length of clearance period.

Clearance period (years)	Total study number	Percentage included	Percentage drop
0	2344254	100.00	0.00
1	1850191	78.92	21.08
2	1551637	66.19	33.81
3	1317400	56.20	43.80
4	1121513	47.84	52.16
5	965905	41.20	58.80
6	842139	35.92	64.08
7	741290	31.62	68.38
8	658740	28.10	71.90
9	587570	25.06	74.94
10	523573	22.33	77.67

Supplementary Figure 2: Multi-state model illustrating the disease pathways and trajectories of chronic disease accrual to death (CDF- Chronic Disease Free).

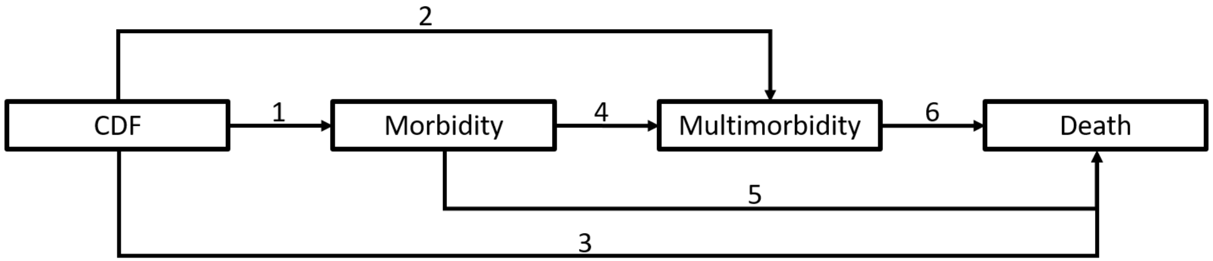

Supplementary Table 3: Restricted Mean Survival Time (RMST) by trajectory and socioeconomic patient group for 5-year clearance with deprivation measured at study start.

Demographic	Trajectory	Most deprived RMST (years)	Second most deprived RMST (years)	Middle deprivation RMST (years)	Second least deprived RMST (years)	Least deprived RMST (years)	Gain (+) or loss (-) in time spent in state for most vs least deprived	99% Confidence Intervals of difference		Gain (+) or loss (-) in time spent in state for second most vs least deprived	99% Confidence Intervals of difference		Gain (+) or loss (-) in time spent in state for middle vs least deprived	99% Confidence Intervals of difference		Gain (+) or loss (-) in time spent in state for second least vs least deprived	99% Confidence Intervals of difference	
10 year old female	CDF -> Morbidity	6.91	6.85	6.82	6.88	7.00	-0.09	-0.09	-0.09	-0.15	-0.15	-0.15	-0.18	-0.18	-0.17	-0.12	-0.12	-0.12
	CDF -> MM	14.66	14.68	14.70	14.70	14.71	-0.05	-0.05	-0.05	-0.03	-0.03	-0.03	-0.01	-0.01	-0.01	-0.01	-0.01	-0.01
	CDF -> Death	14.99	14.99	14.99	14.99	14.99	<-0.01	<-0.01	<-0.01	<-0.01	<-0.01	<-0.01	<-0.01	<-0.01	<-0.01	<-0.01	<-0.01	<-0.01
	Morbidity -> MM	5.37	5.46	5.51	5.52	5.66	-0.30	-0.30	-0.29	-0.21	-0.21	-0.21	-0.16	-0.16	-0.15	-0.14	-0.15	-0.14
	Morbidity -> Death	14.99	14.99	14.99	14.99	14.99	<-0.01	<-0.01	<-0.01	<-0.01	<-0.01	<-0.01	<-0.01	<-0.01	<-0.01	<-0.01	<-0.01	<-0.01
	MM -> Death	14.87	14.88	14.89	14.91	14.91	-0.04	-0.04	-0.04	-0.03	-0.03	-0.03	-0.02	-0.02	-0.02	-0.01	-0.01	-0.01
10 year old Male	CDF -> Morbidity	8.94	8.88	8.86	8.91	9.02	-0.08	-0.08	-0.08	-0.14	-0.14	-0.13	-0.16	-0.16	-0.16	-0.11	-0.11	-0.11
	CDF -> MM	14.74	14.75	14.76	14.76	14.77	-0.04	-0.04	-0.04	-0.02	-0.02	-0.02	-0.01	-0.01	-0.01	-0.01	-0.01	-0.01
	CDF -> Death	14.98	14.99	14.99	14.99	14.99	<-0.01	<-0.01	<-0.01	<-0.01	<-0.01	<-0.01	<-0.01	<-0.01	<-0.01	<-0.01	<-0.01	<-0.01
	Morbidity -> MM	6.44	6.53	6.59	6.60	6.75	-0.30	-0.30	-0.30	-0.21	-0.21	-0.21	-0.16	-0.16	-0.16	-0.15	-0.15	-0.14
	Morbidity -> Death	14.98	14.98	14.98	14.99	14.99	-0.01	-0.01	-0.01	<-0.01	<-0.01	<-0.01	<-0.01	<-0.01	<-0.01	<-0.01	<-0.01	<-0.01
	MM -> Death	14.83	14.85	14.86	14.88	14.89	-0.06	-0.06	-0.06	-0.04	-0.04	-0.04	-0.02	-0.02	-0.02	-0.01	-0.01	-0.01
20 year old female	CDF -> Morbidity	6.89	6.83	6.80	6.85	6.98	-0.09	-0.09	-0.09	-0.15	-0.15	-0.15	-0.18	-0.18	-0.17	-0.12	-0.12	-0.12
	CDF -> MM	14.56	14.58	14.60	14.60	14.62	-0.06	-0.06	-0.06	-0.04	-0.04	-0.04	-0.02	-0.02	-0.01	-0.01	-0.01	-0.01
	CDF -> Death	14.98	14.98	14.98	14.98	14.99	-0.01	-0.01	-0.01	<-0.01	<-0.01	<-0.01	<-0.01	<-0.01	<-0.01	<-0.01	<-0.01	<-0.01
	Morbidity -> MM	4.99	5.07	5.13	5.14	5.28	-0.29	-0.29	-0.29	-0.20	-0.21	-0.20	-0.15	-0.15	-0.15	-0.14	-0.14	-0.14
	Morbidity -> Death	14.98	14.98	14.98	14.98	14.98	-0.01	-0.01	-0.01	-0.01	-0.01	-0.01	<-0.01	<-0.01	<-0.01	<-0.01	<-0.01	<-0.01
	MM -> Death	14.76	14.78	14.81	14.83	14.84	-0.08	-0.08	-0.08	-0.06	-0.06	-0.06	-0.03	-0.04	-0.03	-0.01	-0.02	-0.01
20 year old male	CDF -> Morbidity	8.91	8.86	8.83	8.89	9.00	-0.08	-0.08	-0.08	-0.14	-0.14	-0.13	-0.16	-0.16	-0.16	-0.11	-0.11	-0.11

	CDF -> MM	14.66	14.67	14.69	14.69	14.70	-0.05	-0.05	-0.05	-0.03	-0.03	-0.03	-0.01	-0.01	-0.01	-0.01	-0.01	-0.01
	CDF -> Death	14.98	14.98	14.98	14.98	14.98	-0.01	-0.01	-0.01	-0.01	-0.01	-0.01	<-0.01	<-0.01	<-0.01	<-0.01	<-0.01	<-0.01
	Morbidity -> MM	6.05	6.14	6.19	6.20	6.35	-0.30	-0.30	-0.30	-0.21	-0.21	-0.21	-0.16	-0.16	-0.16	-0.15	-0.15	-0.14
	Morbidity -> Death	14.97	14.97	14.97	14.98	14.98	-0.01	-0.01	-0.01	-0.01	-0.01	-0.01	-0.01	-0.01	-0.01	<-0.01	<-0.01	<-0.01
	MM -> Death	14.69	14.72	14.75	14.77	14.79	-0.11	-0.11	-0.10	-0.08	-0.08	-0.07	-0.05	-0.05	-0.04	-0.02	-0.02	-0.02
30 year old female	CDF -> Morbidity	6.86	6.81	6.78	6.83	6.95	-0.09	-0.09	-0.09	-0.15	-0.15	-0.15	-0.18	-0.18	-0.17	-0.12	-0.12	-0.12
	CDF -> MM	14.42	14.44	14.48	14.48	14.50	-0.08	-0.08	-0.08	-0.05	-0.05	-0.05	-0.02	-0.02	-0.02	-0.02	-0.02	-0.02
	CDF -> Death	14.97	14.97	14.97	14.97	14.98	-0.01	-0.01	-0.01	-0.01	-0.01	-0.01	-0.01	-0.01	-0.01	-0.01	-0.01	-0.01
	Morbidity -> MM	4.62	4.70	4.75	4.76	4.90	-0.28	-0.28	-0.28	-0.20	-0.20	-0.20	-0.15	-0.15	-0.15	-0.14	-0.14	-0.14
	Morbidity -> Death	14.95	14.96	14.96	14.97	14.97	-0.02	-0.02	-0.02	-0.01	-0.01	-0.01	-0.01	-0.01	-0.01	<-0.01	<-0.01	<-0.01
	MM -> Death	14.55	14.59	14.64	14.68	14.70	-0.15	-0.16	-0.15	-0.11	-0.11	-0.11	-0.06	-0.07	-0.06	-0.03	-0.03	-0.03
30 year old male	CDF -> Morbidity	8.89	8.84	8.81	8.86	8.97	-0.08	-0.08	-0.08	-0.14	-0.14	-0.13	-0.16	-0.16	-0.16	-0.11	-0.11	-0.11
	CDF -> MM	14.55	14.57	14.60	14.60	14.61	-0.06	-0.06	-0.06	-0.04	-0.04	-0.04	-0.02	-0.02	-0.02	-0.01	-0.01	-0.01
	CDF -> Death	14.96	14.96	14.97	14.97	14.97	-0.01	-0.02	-0.01	-0.01	-0.01	-0.01	-0.01	-0.01	-0.01	-0.01	-0.01	-0.01
	Morbidity -> MM	5.66	5.75	5.80	5.81	5.96	-0.30	-0.30	-0.30	-0.21	-0.21	-0.21	-0.16	-0.16	-0.16	-0.15	-0.15	-0.14
	Morbidity -> Death	14.93	14.94	14.95	14.96	14.96	-0.03	-0.03	-0.03	-0.02	-0.02	-0.02	-0.01	-0.01	-0.01	<-0.01	<-0.01	<-0.01
	MM -> Death	14.41	14.47	14.53	14.58	14.61	-0.20	-0.20	-0.20	-0.14	-0.14	-0.14	-0.08	-0.09	-0.08	-0.04	-0.04	-0.03
40 year old female	CDF -> Morbidity	6.84	6.78	6.75	6.81	6.93	-0.09	-0.09	-0.09	-0.15	-0.15	-0.15	-0.18	-0.18	-0.17	-0.12	-0.12	-0.12
	CDF -> MM	14.24	14.27	14.32	14.32	14.35	-0.10	-0.10	-0.10	-0.07	-0.07	-0.07	-0.03	-0.03	-0.03	-0.02	-0.02	-0.02
	CDF -> Death	14.94	14.95	14.95	14.96	14.97	-0.02	-0.02	-0.02	-0.02	-0.02	-0.02	-0.01	-0.01	-0.01	-0.01	-0.01	-0.01
	Morbidity -> MM	4.26	4.34	4.39	4.40	4.53	-0.27	-0.28	-0.27	-0.19	-0.19	-0.19	-0.14	-0.15	-0.14	-0.13	-0.13	-0.13
	Morbidity -> Death	14.90	14.92	14.93	14.94	14.95	-0.04	-0.04	-0.04	-0.03	-0.03	-0.03	-0.02	-0.02	-0.02	-0.01	-0.01	-0.01
	MM -> Death	14.16	14.24	14.32	14.39	14.44	-0.28	-0.29	-0.28	-0.20	-0.21	-0.20	-0.12	-0.13	-0.12	-0.05	-0.06	-0.05
40 year old male	CDF -> Morbidity	8.87	8.82	8.79	8.84	8.95	-0.08	-0.08	-0.08	-0.14	-0.14	-0.14	-0.16	-0.16	-0.16	-0.11	-0.11	-0.11
	CDF -> MM	14.41	14.44	14.47	14.47	14.49	-0.08	-0.08	-0.08	-0.06	-0.06	-0.05	-0.02	-0.02	-0.02	-0.02	-0.02	-0.02
	CDF -> Death	14.93	14.94	14.94	14.95	14.96	-0.03	-0.03	-0.03	-0.02	-0.02	-0.02	-0.01	-0.01	-0.01	-0.01	-0.01	-0.01
	Morbidity	5.27	5.36	5.41	5.42	5.57	-0.30	-0.30	-0.29	-0.21	-0.21	-0.21	-0.16	-0.16	-0.15	-0.14	-0.14	-0.14

	-> MM																
	Morbidity -> Death	14.86	14.88	14.89	14.91	14.92	-0.06	-0.06	-0.06	-0.04	-0.04	-0.04	-0.03	-0.03	-0.03	-0.01	-0.01	-0.01
	MM -> Death	13.91	14.01	14.12	14.21	14.28	-0.37	-0.37	-0.36	-0.26	-0.27	-0.26	-0.16	-0.16	-0.15	-0.07	-0.07	-0.06
50 year old female	CDF -> Morbidity	6.82	6.76	6.73	6.78	6.91	-0.09	-0.09	-0.09	-0.15	-0.15	-0.15	-0.18	-0.18	-0.17	-0.12	-0.12	-0.12
	CDF -> MM	14.01	14.05	14.11	14.12	14.15	-0.13	-0.13	-0.13	-0.09	-0.09	-0.09	-0.03	-0.03	-0.03	-0.03	-0.03	-0.03
	CDF -> Death	14.90	14.91	14.92	14.92	14.94	-0.04	-0.04	-0.04	-0.03	-0.03	-0.03	-0.02	-0.02	-0.02	-0.02	-0.02	-0.02
	Morbidity -> MM	3.92	3.99	4.04	4.05	4.18	-0.26	-0.26	-0.26	-0.19	-0.19	-0.18	-0.14	-0.14	-0.14	-0.13	-0.13	-0.13
	Morbidity -> Death	14.79	14.82	14.84	14.87	14.89	-0.10	-0.10	-0.09	-0.07	-0.07	-0.06	-0.04	-0.05	-0.04	-0.02	-0.02	-0.02
	MM -> Death	13.45	13.60	13.75	13.87	13.97	-0.52	-0.53	-0.51	-0.37	-0.38	-0.36	-0.22	-0.23	-0.21	-0.09	-0.10	-0.09
50 year old male	CDF -> Morbidity	8.85	8.80	8.77	8.82	8.93	-0.08	-0.08	-0.08	-0.14	-0.14	-0.14	-0.16	-0.16	-0.16	-0.11	-0.11	-0.11
	CDF -> MM	14.23	14.26	14.31	14.31	14.34	-0.10	-0.10	-0.10	-0.07	-0.07	-0.07	-0.03	-0.03	-0.03	-0.02	-0.02	-0.02
	CDF -> Death	14.87	14.88	14.90	14.90	14.93	-0.05	-0.05	-0.05	-0.04	-0.04	-0.04	-0.03	-0.03	-0.03	-0.02	-0.02	-0.02
	Morbidity -> MM	4.89	4.98	5.03	5.04	5.18	-0.29	-0.29	-0.29	-0.20	-0.20	-0.20	-0.15	-0.15	-0.15	-0.14	-0.14	-0.14
	Morbidity -> Death	14.70	14.75	14.78	14.82	14.84	-0.14	-0.14	-0.14	-0.09	-0.09	-0.09	-0.06	-0.07	-0.06	-0.02	-0.02	-0.02
	MM -> Death	13.00	13.19	13.38	13.54	13.66	-0.66	-0.67	-0.65	-0.47	-0.48	-0.46	-0.28	-0.29	-0.27	-0.12	-0.13	-0.11
60 year old female	CDF -> Morbidity	6.79	6.73	6.71	6.76	6.88	-0.09	-0.09	-0.09	-0.15	-0.15	-0.15	-0.18	-0.18	-0.17	-0.12	-0.12	-0.12
	CDF -> MM	13.72	13.77	13.85	13.85	13.89	-0.17	-0.17	-0.17	-0.12	-0.12	-0.12	-0.04	-0.04	-0.04	-0.04	-0.04	-0.04
	CDF -> Death	14.81	14.83	14.85	14.85	14.89	-0.08	-0.08	-0.08	-0.06	-0.06	-0.06	-0.04	-0.04	-0.04	-0.04	-0.04	-0.04
	Morbidity -> MM	3.59	3.66	3.71	3.72	3.84	-0.25	-0.25	-0.25	-0.18	-0.18	-0.18	-0.13	-0.13	-0.13	-0.12	-0.12	-0.12
	Morbidity -> Death	14.55	14.62	14.66	14.72	14.76	-0.21	-0.21	-0.21	-0.14	-0.14	-0.14	-0.10	-0.10	-0.10	-0.04	-0.04	-0.03
	MM -> Death	12.20	12.46	12.71	12.94	13.10	-0.90	-0.92	-0.89	-0.65	-0.66	-0.63	-0.39	-0.40	-0.38	-0.17	-0.18	-0.15
60 year old male	CDF -> Morbidity	8.83	8.77	8.75	8.80	8.91	-0.08	-0.08	-0.08	-0.14	-0.14	-0.14	-0.16	-0.16	-0.16	-0.11	-0.11	-0.11
	CDF -> MM	14.00	14.04	14.10	14.10	14.13	-0.13	-0.13	-0.13	-0.09	-0.09	-0.09	-0.03	-0.03	-0.03	-0.03	-0.03	-0.03
	CDF -> Death	14.76	14.79	14.81	14.82	14.86	-0.10	-0.10	-0.10	-0.08	-0.08	-0.08	-0.05	-0.05	-0.05	-0.05	-0.05	-0.05
	Morbidity -> MM	4.53	4.61	4.66	4.67	4.81	-0.28	-0.28	-0.28	-0.20	-0.20	-0.20	-0.15	-0.15	-0.15	-0.14	-0.14	-0.14
	Morbidity -> Death	14.36	14.45	14.52	14.61	14.66	-0.30	-0.30	-0.29	-0.20	-0.20	-0.20	-0.14	-0.14	-0.14	-0.05	-0.05	-0.05

	MM -> Death	11.44	11.76	12.08	12.36	12.57	-1.12	-1.14	-1.10	-0.81	-0.83	-0.79	-0.49	-0.50	-0.47	-0.21	-0.23	-0.19
70 year old female	CDF -> Morbidity	6.77	6.71	6.68	6.74	6.86	-0.09	-0.09	-0.09	-0.15	-0.15	-0.15	-0.18	-0.18	-0.17	-0.12	-0.12	-0.12
	CDF -> MM	13.34	13.41	13.50	13.51	13.56	-0.22	-0.22	-0.21	-0.15	-0.15	-0.15	-0.06	-0.06	-0.05	-0.05	-0.05	-0.05
	CDF -> Death	14.65	14.68	14.72	14.73	14.80	-0.15	-0.15	-0.15	-0.12	-0.12	-0.12	-0.07	-0.07	-0.07	-0.07	-0.07	-0.07
	Morbidity -> MM	3.28	3.35	3.39	3.40	3.51	-0.24	-0.24	-0.24	-0.17	-0.17	-0.17	-0.13	-0.13	-0.12	-0.12	-0.12	-0.11
	Morbidity -> Death	14.04	14.18	14.27	14.40	14.48	-0.44	-0.45	-0.44	-0.31	-0.31	-0.30	-0.21	-0.21	-0.21	-0.08	-0.08	-0.08
	MM -> Death	10.15	10.55	10.97	11.34	11.61	-1.46	-1.48	-1.44	-1.06	-1.08	-1.04	-0.64	-0.67	-0.62	-0.28	-0.30	-0.26
70 year old male	CDF -> Morbidity	8.81	8.75	8.73	8.78	8.89	-0.08	-0.08	-0.08	-0.14	-0.14	-0.14	-0.16	-0.16	-0.16	-0.11	-0.11	-0.11
	CDF -> MM	13.70	13.75	13.83	13.83	13.87	-0.17	-0.17	-0.17	-0.12	-0.12	-0.12	-0.04	-0.04	-0.04	-0.04	-0.04	-0.04
	CDF -> Death	14.56	14.60	14.66	14.66	14.75	-0.19	-0.19	-0.19	-0.15	-0.15	-0.15	-0.09	-0.09	-0.09	-0.09	-0.09	-0.09
	Morbidity -> MM	4.17	4.25	4.30	4.31	4.44	-0.27	-0.27	-0.27	-0.19	-0.19	-0.19	-0.14	-0.14	-0.14	-0.13	-0.13	-0.13
	Morbidity -> Death	13.63	13.83	13.96	14.15	14.26	-0.63	-0.63	-0.62	-0.43	-0.43	-0.43	-0.30	-0.30	-0.30	-0.11	-0.11	-0.11
	MM -> Death	9.00	9.47	9.96	10.40	10.73	-1.73	-1.75	-1.70	-1.26	-1.29	-1.23	-0.77	-0.80	-0.74	-0.33	-0.36	-0.31

Supplementary Table 4: Restricted Mean Survival Time (RMST) by trajectory and socioeconomic patient group for 1-year clearance.

Demographic	Trajectory	Most Deprived RMST	Least Deprived RMST	Diff	99% CI lower	99% CI upper
10 year old female	Healthy -> Morbidity	7.61	7.92	-0.32	-0.32	-0.32
	Healthy -> MM	18.46	18.57	-0.11	-0.11	-0.11
	Healthy -> Death	18.99	18.99	<-0.01	<-0.01	<-0.01
	Morbidity -> MM	5.16	5.71	-0.55	-0.55	-0.54
	Morbidity -> Death	18.98	18.99	-0.01	-0.01	-0.01
	MM -> Death	18.80	18.86	-0.07	-0.07	-0.07
10 year old Male	Healthy -> Morbidity	9.90	10.21	-0.31	-0.31	-0.31
	Healthy -> MM	18.57	18.66	-0.09	-0.09	-0.09
	Healthy -> Death	18.99	18.99	<-0.01	<-0.01	<-0.01
	Morbidity -> MM	6.38	6.97	-0.59	-0.59	-0.59
	Morbidity -> Death	18.98	18.98	-0.01	-0.01	-0.01
	MM -> Death	18.74	18.82	-0.09	-0.09	-0.08
20 year old female	Healthy -> Morbidity	7.26	7.57	-0.32	-0.32	-0.32
	Healthy -> MM	18.26	18.41	-0.15	-0.15	-0.15
	Healthy -> Death	18.98	18.98	-0.01	-0.01	-0.01
	Morbidity -> MM	4.60	5.12	-0.52	-0.52	-0.51
	Morbidity -> Death	18.96	18.98	-0.01	-0.01	-0.01
	MM -> Death	18.64	18.76	-0.12	-0.12	-0.12
20 year old male	Healthy -> Morbidity	9.56	9.87	-0.32	-0.32	-0.31
	Healthy -> MM	18.41	18.53	-0.12	-0.12	-0.12
	Healthy -> Death	18.98	18.98	-0.01	-0.01	-0.01
	Morbidity -> MM	5.76	6.34	-0.57	-0.57	-0.57
	Morbidity -> Death	18.95	18.97	-0.02	-0.02	-0.02
	MM -> Death	18.53	18.69	-0.15	-0.15	-0.15
30 year old female	Healthy -> Morbidity	6.91	7.23	-0.31	-0.31	-0.31
	Healthy -> MM	17.97	18.18	-0.21	-0.21	-0.21
	Healthy -> Death	18.96	18.97	-0.01	-0.01	-0.01

	Morbidity -> MM	4.08	4.56	-0.48	-0.48	-0.48
	Morbidity -> Death	18.93	18.95	-0.03	-0.03	-0.03
	MM -> Death	18.35	18.56	-0.21	-0.22	-0.21
30 year old male	Healthy -> Morbidity	9.21	9.52	-0.32	-0.32	-0.32
	Healthy -> MM	18.18	18.35	-0.17	-0.17	-0.17
	Healthy -> Death	18.95	18.97	-0.01	-0.01	-0.01
	Morbidity -> MM	5.17	5.72	-0.55	-0.55	-0.54
	Morbidity -> Death	18.90	18.94	-0.04	-0.04	-0.04
	MM -> Death	18.16	18.43	-0.28	-0.28	-0.28
40 year old female	Healthy -> Morbidity	6.57	6.88	-0.31	-0.31	-0.31
	Healthy -> MM	17.57	17.86	-0.29	-0.29	-0.29
	Healthy -> Death	18.92	18.95	-0.03	-0.03	-0.03
	Morbidity -> MM	3.60	4.04	-0.44	-0.44	-0.44
	Morbidity -> Death	18.84	18.90	-0.06	-0.06	-0.06
	MM -> Death	17.83	18.21	-0.38	-0.38	-0.38
40 year old male	Healthy -> Morbidity	8.85	9.17	-0.32	-0.32	-0.32
	Healthy -> MM	17.87	18.10	-0.23	-0.23	-0.23
	Healthy -> Death	18.91	18.94	-0.03	-0.03	-0.03
	Morbidity -> MM	4.61	5.13	-0.52	-0.52	-0.51
	Morbidity -> Death	18.78	18.87	-0.08	-0.08	-0.08
	MM -> Death	17.49	17.98	-0.49	-0.49	-0.49
50 year old female	Healthy -> Morbidity	6.24	6.54	-0.30	-0.30	-0.30
	Healthy -> MM	17.04	17.43	-0.39	-0.39	-0.39
	Healthy -> Death	18.84	18.90	-0.06	-0.06	-0.06
	Morbidity -> MM	3.16	3.56	-0.40	-0.40	-0.40
	Morbidity -> Death	18.63	18.77	-0.15	-0.15	-0.15
	MM -> Death	16.93	17.59	-0.67	-0.67	-0.66
50 year old male	Healthy -> Morbidity	8.50	8.82	-0.32	-0.32	-0.32
	Healthy -> MM	17.43	17.75	-0.32	-0.32	-0.31
	Healthy -> Death	18.81	18.88	-0.07	-0.07	-0.07
	Morbidity -> MM	4.09	4.57	-0.48	-0.48	-0.48

	Morbidity -> Death	18.50	18.70	-0.20	-0.20	-0.19
	MM -> Death	16.35	17.19	-0.84	-0.84	-0.84
60 year old female	Healthy -> Morbidity	5.92	6.21	-0.30	-0.30	-0.30
	Healthy -> MM	16.32	16.85	-0.52	-0.52	-0.52
	Healthy -> Death	18.66	18.78	-0.13	-0.13	-0.13
	Morbidity -> MM	2.76	3.13	-0.36	-0.36	-0.36
	Morbidity -> Death	18.15	18.48	-0.34	-0.34	-0.33
	MM -> Death	15.40	16.52	-1.12	-1.12	-1.11
60 year old male	Healthy -> Morbidity	8.15	8.47	-0.32	-0.32	-0.32
	Healthy -> MM	16.85	17.28	-0.43	-0.43	-0.43
	Healthy -> Death	18.61	18.75	-0.15	-0.15	-0.14
	Morbidity -> MM	3.60	4.05	-0.44	-0.44	-0.44
	Morbidity -> Death	17.87	18.32	-0.44	-0.44	-0.44
	MM -> Death	14.47	15.84	-1.38	-1.38	-1.37
70 year old female	Healthy -> Morbidity	5.60	5.89	-0.29	-0.29	-0.29
	Healthy -> MM	15.38	16.07	-0.69	-0.69	-0.68
	Healthy -> Death	18.28	18.55	-0.27	-0.27	-0.27
	Morbidity -> MM	2.41	2.73	-0.32	-0.32	-0.32
	Morbidity -> Death	17.08	17.82	-0.74	-0.75	-0.74
	MM -> Death	12.99	14.74	-1.75	-1.75	-1.74
70 year old male	Healthy -> Morbidity	7.80	8.12	-0.32	-0.32	-0.32
	Healthy -> MM	16.07	16.64	-0.57	-0.57	-0.57
	Healthy -> Death	18.17	18.48	-0.31	-0.31	-0.30
	Morbidity -> MM	3.16	3.57	-0.40	-0.40	-0.40
	Morbidity -> Death	16.49	17.45	-0.96	-0.96	-0.96
	MM -> Death	11.61	13.66	-2.05	-2.06	-2.05
*max RMST = 19 years						

Supplementary Table 5: Restricted Mean Survival Time (RMST) by trajectory and socioeconomic patient group for 10-year clearance.

Demographic	Trajectory	Most deprived RMST (years)	Second most deprived RMST (years)	Middle deprivation RMST (years)	Second least deprived RMST (years)	Least deprived RMST (years)	Gain (+) or loss (-) in time spent in state for most vs least deprived	99% Confidence Intervals of difference		Gain (+) or loss (-) in time spent in state for second most vs least deprived	99% Confidence Intervals of difference		Gain (+) or loss (-) in time spent in state for middle vs least deprived	99% Confidence Intervals of difference		Gain (+) or loss (-) in time spent in state for second least vs least deprived	99% Confidence Intervals of difference	
20 year old female	CDF -> Morbidity	5.89	5.88	5.87	5.91	5.98	-0.09	-0.09	-0.09	-0.10	-0.10	-0.10	-0.12	-0.12	-0.11	-0.07	-0.08	-0.07
	CDF -> MM	9.83	9.83	9.84	9.85	9.85	-0.03	-0.03	-0.03	-0.02	-0.02	-0.02	-0.01	-0.01	-0.01	-0.01	-0.01	-0.01
	CDF -> Death	9.99	9.99	9.99	9.99	9.99	<-0.01	<-0.01	<-0.01	<-0.01	<-0.01	<-0.01	<-0.01	<-0.01	<-0.01	<-0.01	<-0.01	<-0.01
	Morbidity -> MM	4.69	4.80	4.88	4.94	5.08	-0.40	-0.40	-0.39	-0.28	-0.28	-0.28	-0.21	-0.21	-0.20	-0.14	-0.15	-0.14
	Morbidity -> Death	9.99	9.99	9.99	9.99	9.99	-0.01	-0.01	-0.01	<-0.01	<-0.01	<-0.01	<-0.01	<-0.01	<-0.01	<-0.01	<-0.01	<-0.01
	MM -> Death	9.86	9.87	9.89	9.90	9.91	-0.05	-0.05	-0.05	-0.04	-0.04	-0.04	-0.02	-0.02	-0.02	-0.01	-0.01	-0.01
20 year old male	CDF -> Morbidity	7.16	7.15	7.14	7.17	7.23	-0.07	-0.07	-0.07	-0.08	-0.08	-0.08	-0.09	-0.09	-0.09	-0.06	-0.06	-0.06
	CDF -> MM	9.86	9.87	9.88	9.88	9.89	-0.02	-0.02	-0.02	-0.02	-0.02	-0.02	-0.01	-0.01	-0.01	<-0.01	<-0.01	<-0.01
	CDF -> Death	9.99	9.99	9.99	9.99	9.99	<-0.01	<-0.01	<-0.01	<-0.01	<-0.01	<-0.01	<-0.01	<-0.01	<-0.01	<-0.01	<-0.01	<-0.01
	Morbidity -> MM	5.38	5.49	5.56	5.62	5.76	-0.38	-0.38	-0.38	-0.27	-0.27	-0.26	-0.20	-0.20	-0.19	-0.14	-0.14	-0.13
	Morbidity -> Death	9.98	9.98	9.99	9.99	9.99	-0.01	-0.01	-0.01	-0.01	-0.01	-0.01	<-0.01	<-0.01	<-0.01	<-0.01	<-0.01	<-0.01
	MM -> Death	9.82	9.83	9.86	9.87	9.89	-0.07	-0.07	-0.07	-0.06	-0.06	-0.05	-0.03	-0.03	-0.03	-0.02	-0.02	-0.02
30 year old female	CDF -> Morbidity	5.94	5.93	5.91	5.96	6.03	-0.09	-0.09	-0.09	-0.10	-0.10	-0.10	-0.12	-0.12	-0.11	-0.07	-0.07	-0.07
	CDF -> MM	9.77	9.78	9.80	9.80	9.81	-0.04	-0.04	-0.04	-0.03	-0.03	-0.03	-0.01	-0.01	-0.01	-0.01	-0.01	-0.01
	CDF -> Death	9.98	9.98	9.99	9.99	9.99	-0.01	-0.01	-0.01	<-0.01	<-0.01	<-0.01	<-0.01	<-0.01	<-0.01	<-0.01	<-0.01	<-0.01
	Morbidity	4.38	4.50	4.57	4.64	4.78	-0.40	-0.40	-0.40	-0.28	-0.29	-0.28	-0.21	-0.21	-0.21	-0.14	-0.15	-0.14

	-> MM																
	Morbidity -> Death	9.97	9.98	9.98	9.98	9.99	-0.01	-0.01	-0.01	-0.01	-0.01	-0.01	<-0.01	<-0.01	<-0.01	<-0.01	<-0.01	<-0.01
	MM -> Death	9.73	9.75	9.79	9.81	9.83	-0.10	-0.10	-0.10	-0.08	-0.08	-0.08	-0.05	-0.05	-0.05	-0.02	-0.03	-0.02
30 year old male	CDF -> Morbidity	7.20	7.19	7.18	7.21	7.27	-0.07	-0.07	-0.07	-0.08	-0.08	-0.08	-0.09	-0.09	-0.09	-0.06	-0.06	-0.06
	CDF -> MM	9.82	9.83	9.84	9.84	9.85	-0.03	-0.03	-0.03	-0.02	-0.02	-0.02	-0.01	-0.01	-0.01	-0.01	-0.01	-0.01
	CDF -> Death	9.98	9.98	9.98	9.98	9.99	-0.01	-0.01	-0.01	-0.01	-0.01	-0.01	<-0.01	<-0.01	<-0.01	<-0.01	<-0.01	<-0.01
	Morbidity -> MM	5.09	5.20	5.27	5.33	5.47	-0.39	-0.39	-0.38	-0.27	-0.28	-0.27	-0.20	-0.20	-0.20	-0.14	-0.14	-0.14
	Morbidity -> Death	9.97	9.97	9.98	9.98	9.98	-0.02	-0.02	-0.02	-0.01	-0.01	-0.01	-0.01	-0.01	-0.01	-0.01	-0.01	-0.01
	MM -> Death	9.66	9.69	9.73	9.76	9.79	-0.13	-0.13	-0.13	-0.11	-0.11	-0.10	-0.06	-0.06	-0.06	-0.03	-0.03	-0.03
40 year old female	CDF -> Morbidity	5.99	5.98	5.96	6.00	6.07	-0.09	-0.09	-0.09	-0.10	-0.10	-0.10	-0.11	-0.12	-0.11	-0.07	-0.07	-0.07
	CDF -> MM	9.70	9.71	9.73	9.74	9.75	-0.05	-0.05	-0.05	-0.03	-0.04	-0.03	-0.02	-0.02	-0.02	-0.01	-0.01	-0.01
	CDF -> Death	9.97	9.97	9.98	9.98	9.98	-0.01	-0.01	-0.01	-0.01	-0.01	-0.01	<-0.01	<-0.01	<-0.01	<-0.01	<-0.01	<-0.01
	Morbidity -> MM	4.08	4.19	4.27	4.33	4.48	-0.40	-0.40	-0.40	-0.28	-0.29	-0.28	-0.21	-0.21	-0.21	-0.14	-0.15	-0.14
	Morbidity -> Death	9.95	9.96	9.96	9.97	9.98	-0.03	-0.03	-0.03	-0.02	-0.02	-0.02	-0.01	-0.01	-0.01	-0.01	-0.01	-0.01
	MM -> Death	9.49	9.53	9.60	9.64	9.69	-0.20	-0.20	-0.19	-0.16	-0.16	-0.16	-0.09	-0.09	-0.09	-0.05	-0.05	-0.05
40 year old male	CDF -> Morbidity	7.24	7.23	7.22	7.25	7.31	-0.07	-0.07	-0.07	-0.08	-0.08	-0.08	-0.09	-0.09	-0.09	-0.06	-0.06	-0.06
	CDF -> MM	9.77	9.78	9.79	9.80	9.80	-0.04	-0.04	-0.04	-0.03	-0.03	-0.03	-0.01	-0.01	-0.01	-0.01	-0.01	-0.01
	CDF -> Death	9.96	9.97	9.97	9.98	9.98	-0.01	-0.01	-0.01	-0.01	-0.01	-0.01	-0.01	-0.01	-0.01	<-0.01	<-0.01	<-0.01
	Morbidity -> MM	4.79	4.90	4.97	5.04	5.18	-0.39	-0.40	-0.39	-0.28	-0.28	-0.28	-0.20	-0.21	-0.20	-0.14	-0.14	-0.14
	Morbidity -> Death	9.93	9.94	9.95	9.96	9.97	-0.04	-0.04	-0.04	-0.03	-0.03	-0.03	-0.01	-0.01	-0.01	-0.01	-0.01	-0.01
	MM -> Death	9.36	9.41	9.49	9.55	9.61	-0.25	-0.25	-0.25	-0.20	-0.20	-0.20	-0.11	-0.11	-0.11	-0.06	-0.06	-0.06
50 year old female	CDF -> Morbidity	6.03	6.02	6.01	6.05	6.12	-0.09	-0.09	-0.09	-0.10	-0.10	-0.10	-0.11	-0.11	-0.11	-0.07	-0.07	-0.07
	CDF -> MM	9.61	9.63	9.65	9.66	9.67	-0.06	-0.06	-0.06	-0.05	-0.05	-0.05	-0.02	-0.02	-0.02	-0.01	-0.01	-0.01

	CDF -> Death	9.95	9.96	9.96	9.97	9.97	-0.02	-0.02	-0.02	-0.01	-0.01	-0.01	-0.01	-0.01	-0.01	<-0.01	<-0.01	<-0.01
	Morbidity -> MM	3.78	3.89	3.97	4.03	4.17	-0.40	-0.40	-0.39	-0.28	-0.29	-0.28	-0.21	-0.21	-0.21	-0.14	-0.15	-0.14
	Morbidity -> Death	9.89	9.91	9.93	9.93	9.95	-0.06	-0.06	-0.06	-0.04	-0.04	-0.04	-0.02	-0.02	-0.02	-0.02	-0.02	-0.02
	MM -> Death	9.04	9.11	9.24	9.32	9.41	-0.37	-0.37	-0.36	-0.29	-0.30	-0.29	-0.17	-0.17	-0.16	-0.09	-0.09	-0.08
50 year old male	CDF -> Morbidity	7.27	7.26	7.25	7.28	7.34	-0.07	-0.07	-0.07	-0.08	-0.08	-0.08	-0.09	-0.09	-0.09	-0.06	-0.06	-0.06
	CDF -> MM	9.69	9.71	9.72	9.73	9.74	-0.05	-0.05	-0.05	-0.04	-0.04	-0.04	-0.02	-0.02	-0.02	-0.01	-0.01	-0.01
	CDF -> Death	9.94	9.94	9.95	9.96	9.96	-0.02	-0.02	-0.02	-0.02	-0.02	-0.02	-0.01	-0.01	-0.01	<-0.01	<-0.01	<-0.01
	Morbidity -> MM	4.48	4.60	4.67	4.74	4.88	-0.40	-0.40	-0.40	-0.28	-0.29	-0.28	-0.21	-0.21	-0.20	-0.14	-0.15	-0.14
	Morbidity -> Death	9.85	9.87	9.90	9.91	9.94	-0.08	-0.08	-0.08	-0.06	-0.06	-0.06	-0.03	-0.03	-0.03	-0.03	-0.03	-0.03
	MM -> Death	8.80	8.89	9.05	9.14	9.25	-0.45	-0.46	-0.45	-0.37	-0.37	-0.36	-0.21	-0.21	-0.20	-0.11	-0.11	-0.11
60 year old female	CDF -> Morbidity	6.08	6.07	6.05	6.09	6.17	-0.09	-0.09	-0.09	-0.10	-0.10	-0.10	-0.11	-0.11	-0.11	-0.07	-0.07	-0.07
	CDF -> MM	9.49	9.51	9.54	9.55	9.57	-0.08	-0.08	-0.08	-0.06	-0.06	-0.06	-0.03	-0.03	-0.03	-0.02	-0.02	-0.02
	CDF -> Death	9.92	9.93	9.94	9.95	9.95	-0.03	-0.03	-0.03	-0.03	-0.03	-0.03	-0.01	-0.01	-0.01	<-0.01	<-0.01	<-0.01
	Morbidity -> MM	3.48	3.59	3.67	3.73	3.87	-0.39	-0.39	-0.39	-0.28	-0.28	-0.28	-0.21	-0.21	-0.20	-0.14	-0.15	-0.14
	Morbidity -> Death	9.76	9.80	9.85	9.85	9.90	-0.14	-0.14	-0.13	-0.10	-0.10	-0.10	-0.05	-0.05	-0.05	-0.05	-0.05	-0.04
	MM -> Death	8.24	8.36	8.59	8.73	8.89	-0.65	-0.66	-0.65	-0.53	-0.53	-0.52	-0.30	-0.31	-0.30	-0.16	-0.16	-0.15
60 year old male	CDF -> Morbidity	7.31	7.30	7.29	7.32	7.38	-0.07	-0.07	-0.07	-0.08	-0.08	-0.08	-0.09	-0.09	-0.09	-0.06	-0.06	-0.06
	CDF -> MM	9.60	9.62	9.64	9.65	9.66	-0.06	-0.06	-0.06	-0.05	-0.05	-0.05	-0.02	-0.02	-0.02	-0.01	-0.01	-0.01
	CDF -> Death	9.90	9.90	9.92	9.93	9.94	-0.04	-0.04	-0.04	-0.04	-0.04	-0.04	-0.02	-0.02	-0.02	-0.01	-0.01	<-0.01
	Morbidity -> MM	4.18	4.29	4.37	4.43	4.58	-0.40	-0.40	-0.40	-0.28	-0.29	-0.28	-0.21	-0.21	-0.21	-0.14	-0.15	-0.14
	Morbidity -> Death	9.68	9.73	9.79	9.80	9.86	-0.19	-0.19	-0.18	-0.13	-0.14	-0.13	-0.07	-0.07	-0.07	-0.06	-0.06	-0.06
	MM -> Death	7.82	7.97	8.24	8.42	8.61	-0.80	-0.80	-0.79	-0.65	-0.65	-0.64	-0.37	-0.37	-0.36	-0.20	-0.20	-0.19
70 year old female	CDF -> Morbidity	6.12	6.11	6.10	6.14	6.21	-0.09	-0.09	-0.09	-0.10	-0.10	-0.10	-0.11	-0.11	-0.11	-0.07	-0.07	-0.07

	CDF -> MM	9.33	9.36	9.40	9.42	9.44	-0.10	-0.10	-0.10	-0.08	-0.08	-0.08	-0.04	-0.04	-0.04	-0.02	-0.02	-0.02
	CDF -> Death	9.87	9.87	9.90	9.92	9.92	-0.06	-0.06	-0.06	-0.05	-0.05	-0.05	-0.02	-0.02	-0.02	-0.01	-0.01	-0.01
	Morbidity -> MM	3.19	3.30	3.37	3.43	3.57	-0.38	-0.38	-0.38	-0.27	-0.28	-0.27	-0.20	-0.20	-0.20	-0.14	-0.14	-0.14
	Morbidity -> Death	9.49	9.57	9.67	9.68	9.78	-0.30	-0.30	-0.29	-0.21	-0.22	-0.21	-0.11	-0.12	-0.11	-0.10	-0.10	-0.10
	MM -> Death	6.88	7.09	7.46	7.70	7.97	-1.09	-1.10	-1.08	-0.88	-0.89	-0.88	-0.51	-0.52	-0.50	-0.27	-0.28	-0.26
70 year old male	Healthy -> Morbidity	7.34	7.34	7.32	7.36	7.41	-0.07	-0.07	-0.07	-0.08	-0.08	-0.08	-0.09	-0.09	-0.09	-0.06	-0.06	-0.06
	Healthy -> MM	9.48	9.50	9.53	9.54	9.56	-0.08	-0.08	-0.08	-0.06	-0.06	-0.06	-0.03	-0.03	-0.03	-0.02	-0.02	-0.02
	Healthy -> Death	9.82	9.83	9.87	9.89	9.90	-0.07	-0.07	-0.07	-0.06	-0.06	-0.06	-0.03	-0.03	-0.03	-0.01	-0.01	-0.01
	Morbidity -> MM	3.87	3.99	4.07	4.13	4.27	-0.40	-0.40	-0.40	-0.28	-0.29	-0.28	-0.21	-0.21	-0.21	-0.14	-0.15	-0.14
	Morbidity -> Death	9.30	9.41	9.55	9.57	9.70	-0.40	-0.40	-0.40	-0.29	-0.29	-0.29	-0.15	-0.16	-0.15	-0.14	-0.14	-0.13
	MM -> Death	6.23	6.46	6.90	7.18	7.50	-1.27	-1.28	-1.26	-1.04	-1.05	-1.03	-0.60	-0.61	-0.59	-0.32	-0.33	-0.31

Supplementary Table 6: Top three most common first conditions by age-sex groups for individuals living in the most and least deprived areas.

Age group and sex	Most common first condition	Count	Age group and sex	Most common first condition	Count
10-19 year old female in most deprived area	Dermatitis	2853	10-19 year old female in least deprived area	Dermatitis	2140
	Menorrhagia and polymenorrhoea	1519		Allergic and chronic rhinitis	1130
	Dysmenorrhoea	1410		Dysmenorrhoea	1088
10-19 year old male in most deprived area	Dermatitis	1837	10-19 year old male in least deprived area	Dermatitis	1577
	Allergic and chronic rhinitis	1428		Allergic and chronic rhinitis	1368
	Asthma	1012		Asthma	582
20-29 year old female in most deprived area	Cancer	2390	20-29 year old female in least deprived area	Cancer	2020
	Dermatitis	2100		Dermatitis	1482
	Migraine	964		Allergic and chronic rhinitis	800
20-29 year old male in most deprived area	Dermatitis	2034	20-29 year old male in least deprived area	Dermatitis	1524
	Allergic and chronic rhinitis	1273		Allergic and chronic rhinitis	1088
	Other psychoactive substance misuse	1223		Enthesopathies & synovial disorders	739
30-39 year old female in most deprived area	Cancer	1402	30-39 year old female in least deprived area	Cancer	1507
	Dermatitis	1161		Dermatitis	1134
	Menorrhagia and polymenorrhoea	822		Menorrhagia and polymenorrhoea	582
30-39 year old male in most deprived area	Enthesopathies & synovial disorders	1444	30-39 year old male in least deprived area	Dermatitis	1343
	Dermatitis	1423		Enthesopathies & synovial disorders	1279
	Gastro-oesoph disorder	854		Allergic and chronic rhinitis	908
40-49 year old female in most deprived area	Menorrhagia and polymenorrhoea	1215	40-49 year old female in least deprived area	Cancer	1863
	Cancer	1212		Menorrhagia and polymenorrhoea	1293
	Enthesopathies & synovial disorders	1028		Enthesopathies & synovial disorders	1061
40-49 year old male in most deprived area	Enthesopathies & synovial disorders	1906	40-49 year old male in least deprived area	Enthesopathies & synovial disorders	2116
	Hypertension	1621		Dermatitis	1315
	Dermatitis	1172		Hypertension	1301
	Hypertension	999	50-59 year old female in least deprived area	Cancer	1524
	Cancer	799		Enthesopathies & synovial disorders	1050

50-59 year old female in most deprived area	Enthesopathies & synovial disorders	732		Hypertension	927
50-59 year old male in most deprived area	Hypertension	1958	50-59 year old male in least deprived area	Hypertension	1949
	Enthesopathies & synovial disorders	1163		Enthesopathies & synovial disorders	1601
	Dermatitis	685		Dermatitis	903
60-69 year old female in most deprived area	Hypertension	1076	60-69 year old female in least deprived area	Hypertension	1112
	Cancer	495		Cancer	895
	Osteoarthritis (excl spine)	433		Osteoarthritis (excl spine)	545
60-69 year old male in most deprived area	Hypertension	1296	60-69 year old male in least deprived area	Hypertension	1429
	Cancer	453		Enthesopathies & synovial disorders	615
	Osteoarthritis (excl spine)	416		Cancer	605
70-79 year old female in most deprived area	Hypertension	801	70-79 year old female in least deprived area	Hypertension	781
	Osteoarthritis (excl spine)	264		Osteoarthritis (excl spine)	362
	Cataract	218		Cataract	281
70-79 year old male in most deprived area	Hypertension	566	70-79 year old male in least deprived area	Hypertension	564
	Cancer	285		Cancer	330
	Coronary heart disease	191		Hyperplasia of prostate	184

Supplementary Table 7: Top three most common mulitmorbidity by age-sex groups for individuals living in the most and least deprived areas.

Age group and sex	Most common multimorbidity		Count	Age group and sex	Most common multimorbidity		Count
10-19 year old female in most deprived area	Allergic and chronic rhinitis	Dermatitis	274	10-19 year old female in least deprived area	Allergic and chronic rhinitis	Dermatitis	227
	Dermatitis	Menorrhagia and polymenorrhoea	264		Dermatitis	Dysmenorrhoea	162
	Dysmenorrhoea	Menorrhagia and polymenorrhoea	260		Dermatitis	Menorrhagia and polymenorrhoea	139
10-19 year old male in most deprived area	Allergic and chronic rhinitis	Dermatitis	185	10-19 year old male in least deprived area	Allergic and chronic rhinitis	Dermatitis	211
	Allergic and chronic rhinitis	Chronic sinusitis	137		Allergic and chronic rhinitis	Chronic sinusitis	179
	Asthma	Dermatitis	135		Asthma	Dermatitis	97
20-29 year old female in most deprived area	Dermatitis	Menorrhagia and polymenorrhoea	233	20-29 year old female in least deprived area	Dermatitis	Cancer	386
	Dermatitis	Migraine	229		Allergic and chronic rhinitis	Dermatitis	174
	Allergic and chronic rhinitis	Dermatitis	222		Allergic and chronic rhinitis	Cancer	149
20-29 year old male in most deprived area	Alcohol Problems	Other psychoactive substance misuse	229	20-29 year old male in least deprived area	Allergic and chronic rhinitis	Dermatitis	169
	Allergic and chronic rhinitis	Dermatitis	203		Allergic and chronic rhinitis	Chronic sinusitis	159
	Allergic and chronic rhinitis	Chronic sinusitis	162		Dermatitis	Enthesopathies & synovial disorders	116
	Dermatitis	Cancer	234		Dermatitis	Cancer	274

30-39 year old female in most deprived area	Menorrhagia and polymenorrhoea	Cancer	153	30-39 year old female in least deprived area	Migraine	Cancer	113
	Migraine	Cancer	109		Menorrhagia and polymenorrhoea	Cancer	111
30-39 year old male in most deprived area	Dermatitis	Enthesopathies & synovial disorders	200	30-39 year old male in least deprived area	Dermatitis	Enthesopathies & synovial disorders	164
	Alcohol Problems	Other psychoactive substance misuse	175		Dermatitis	Enthesopathies & synovial disorders	164
	Allergic and chronic rhinitis	Dermatitis	103		Allergic and chronic rhinitis	Dermatitis	103
40-49 year old female in most deprived area	Menorrhagia and polymenorrhoea	Cancer	178	40-49 year old female in least deprived area	Menorrhagia and polymenorrhoea	Cancer	252
	Enthesopathies & synovial disorders	Menorrhagia and polymenorrhoea	165		Dermatitis	Cancer	242
	Enthesopathies & synovial disorders	Cancer	156		Enthesopathies & synovial disorders	Cancer	218
40-49 year old male in most deprived area	Dermatitis	Enthesopathies & synovial disorders	222	40-49 year old male in least deprived area	Dermatitis	Enthesopathies & synovial disorders	267
	Enthesopathies & synovial disorders	Hypertension	195		Enthesopathies & synovial disorders	Hypertension	169
	Diabetes	Hypertension	172		Enthesopathies & synovial disorders	Gastro-oesoph disorder	141
50-59 year old female in most deprived area	Enthesopathies & synovial disorders	Cancer	124	50-59 year old female in least deprived area	Enthesopathies & synovial disorders	Cancer	227

	Dermatitis	Enthesopathies & synovial disorders	123		Dermatitis	Cancer	182
	Dermatitis	Cancer	122		Dermatitis	Enthesopathies & synovial disorders	168
50-59 year old male in most deprived area	Enthesopathies & synovial disorders	Hypertension	245	50-59 year old male in least deprived area	Enthesopathies & synovial disorders	Hypertension	277
	Diabetes	Hypertension	241		Dermatitis	Enthesopathies & synovial disorders	226
	Enthesopathies & synovial disorders	Osteoarthritis (excl spine)	171		Enthesopathies & synovial disorders	Osteoarthritis (excl spine)	177
60-69 year old female in most deprived area	Hypertension	Osteoarthritis (excl spine)	120	60-69 year old female in least deprived area	Hypertension	Cancer	155
	Hypertension	Cancer	82		Dermatitis	Hypertension	119
	Enthesopathies & synovial disorders	Hypertension	81		Hypertension	Osteoarthritis (excl spine)	115
60-69 year old male in most deprived area	Diabetes	Hypertension	168	60-69 year old male in least deprived area	Enthesopathies & synovial disorders	Hypertension	163
	Enthesopathies & synovial disorders	Hypertension	134		Hypertension	Osteoarthritis (excl spine)	150
	Hypertension	Osteoarthritis (excl spine)	122		Diabetes	Hypertension	125
70-79 year old female in most deprived area	Hypertension	Osteoarthritis (excl spine)	101	70-79 year old female in least deprived area	Hypertension	Osteoarthritis (excl spine)	109
	CKD	Hypertension	82		Cataract	Hypertension	92
	Cataract	Hypertension	72		CKD	Hypertension	71
70-79 year old male in most deprived area	CKD	Hypertension	54	70-79 year old male in least deprived area	Hypertension	Osteoarthritis (excl spine)	67
	Hypertension	Osteoarthritis (excl spine)	52		Hypertension	Cancer	61

	Hypertension	Cancer	51		Diabetes	Hypertension	56

Supplementary Table 8: Top three most common underlying causes of death by age-sex groups for individuals living in the most and least deprived areas.

Age group and sex	Underlying cause of death	Count	Age group and sex	Underlying cause of death	Count
10-19 year old female in most deprived area	V01-V99 Transport accidents	10	10-19 year old female in least deprived area	C00-C97 Cancer	<5
	X00-X85 Exposure and contact injuries, poisonings, self-harm and assaults	<5		V01-V99 Transport accidents	<5
	C00-C97 Cancer	<5		Q00-Q99 Congenital malformations and chromosomal abnormalities	<5
10-19 year old male in most deprived area	V01-V99 Transport accidents	20	10-19 year old male in least deprived area	V01-V99 Transport accidents	8
	X00-X85 Exposure and contact injuries, poisonings, self-harm and assaults	19		X00-X85 Exposure and contact injuries, poisonings, self-harm and assaults	6
	U00-U85 provisional assignment and emergency use codes	9		C00-C97 Cancer	5
20-29 year old female in most deprived area	X00-X85 Exposure and contact injuries, poisonings, self-harm and assaults	17	20-29 year old female in least deprived area	G00-G99 Disease of nervous system	<5
	C00-C97 Cancer	10		C00-C97 Cancer	<5
	Y00-Y98 Assaults, undetermined intent, operations of war, complications and sequelae of injuries	5		V01-V99 Transport accidents	<5
20-29 year old male in most deprived area	X00-X85 Exposure and contact injuries, poisonings, self-harm and assaults	101	20-29 year old male in least deprived area	X00-X85 Exposure and contact injuries, poisonings, self-harm and assaults	27

	V01-V99 Transport accidents	33		V01-V99 Transport accidents	12
	Y00-Y98 Assaults, undetermined intent, operations of war, complications and sequelae of injuries	16		C00-C97 Cancer	8
30-39 year old female in most deprived area	C00-C97 Cancer	38	30-39 year old female in least deprived area	C00-C97 Cancer	21
	I00-I99 Circulatory system	18		I00-I99 Circulatory system	<5
	K00-K93 Digestive system	15		X00-X85 Exposure and contact injuries, poisonings, self-harm and assaults	<5
30-39 year old male in most deprived area	X00-X85 Exposure and contact injuries, poisonings, self-harm and assaults	88	30-39 year old male in least deprived area	X00-X85 Exposure and contact injuries, poisonings, self-harm and assaults	24
	I00-I99 Circulatory system	45		I00-I99 Circulatory system	15
	C00-C97 Cancer	27		C00-C97 Cancer	14
40-49 year old female in most deprived area	C00-C97 Cancer	107	40-49 year old female in least deprived area	C00-C97 Cancer	74
	I00-I99 Circulatory system	50		I00-I99 Circulatory system	18
	K00-K93 Digestive system	43		V01-V99 Transport accidents	7
40-49 year old male in most deprived area	I00-I99 Circulatory system	170	40-49 year old male in least deprived area	C00-C97 Cancer	78
	C00-C97 Cancer	112		I00-I99 Circulatory system	57
	X00-X85 Exposure and contact injuries, poisonings, self-harm and assaults	97		X00-X85 Exposure and contact injuries, poisonings, self-harm and assaults	36
50-59 year old female in most deprived area	C00-C97 Cancer	297	50-59 year old female in least deprived area	C00-C97 Cancer	204
	I00-I99 Circulatory system	102		I00-I99 Circulatory system	35
	J00-J99 Respiratory system	50		K00-K93 Digestive system	30

50-59 year old male in most deprived area	C00-C97 Cancer	385	50-59 year old male in least deprived area	C00-C97 Cancer	206
	I00-I99 Circulatory system	334		I00-I99 Circulatory system	145
	K00-K93 Digestive system	101		K00-K93 Digestive system	41
60-69 year old female in most deprived area	C00-C97 Cancer	475	60-69 year old female in least deprived area	C00-C97 Cancer	354
	I00-I99 Circulatory system	152		I00-I99 Circulatory system	99
	J00-J99 Respiratory system	116		J00-J99 Respiratory system	38
60-69 year old male in most deprived area	C00-C97 Cancer	701	60-69 year old male in least deprived area	C00-C97 Cancer	507
	I00-I99 Circulatory system	469		I00-I99 Circulatory system	255
	J00-J99 Respiratory system	161		J00-J99 Respiratory system	53
70-79 year old female in most deprived area	C00-C97 Cancer	538	70-79 year old female in least deprived area	C00-C97 Cancer	404
	I00-I99 Circulatory system	299		I00-I99 Circulatory system	187
	J00-J99 Respiratory system	212		J00-J99 Respiratory system	75
70-79 year old male in most deprived area	C00-C97 Cancer	743	70-79 year old male in least deprived area	C00-C97 Cancer	524
	I00-I99 Circulatory system	518		I00-I99 Circulatory system	338
	J00-J99 Respiratory system	240		J00-J99 Respiratory system	104

Supplementary Figure 3: Restricted Mean Survival Time (RMST) by trajectory and socioeconomic patient group for 5-year clearance.

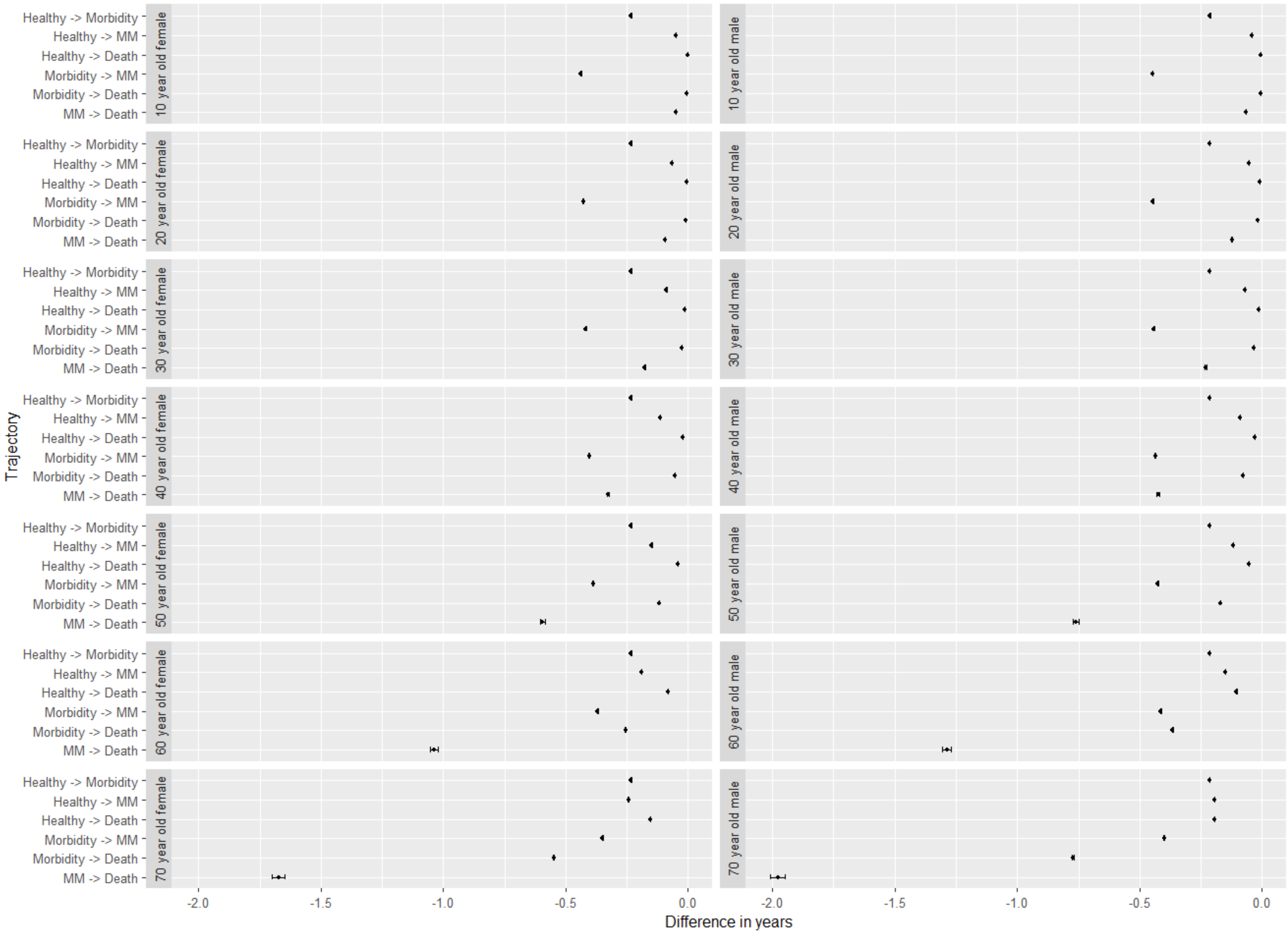

Supplementary Figure 4: Comparison of the cumulative incidence of multimorbidity and mortality between a 20 year old living in one of the most deprived areas of Wales and a 20 year old living in one of the least deprived areas of Wales by sex.

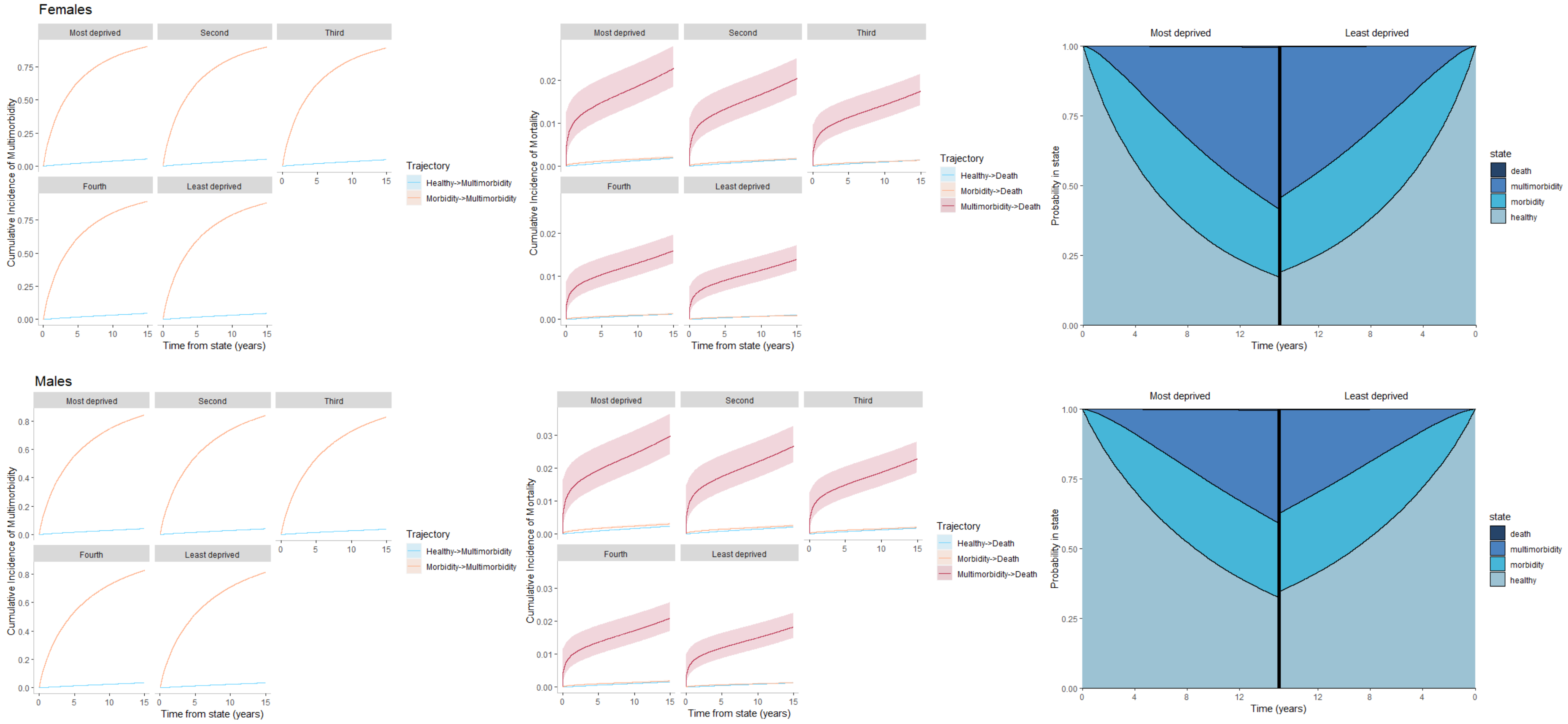

Supplementary Figure 5: Comparison of the cumulative incidence of multimorbidity and mortality between a 30 year old living in one of the most deprived areas of Wales and a 30 year old living in one of the least deprived areas of Wales by sex.

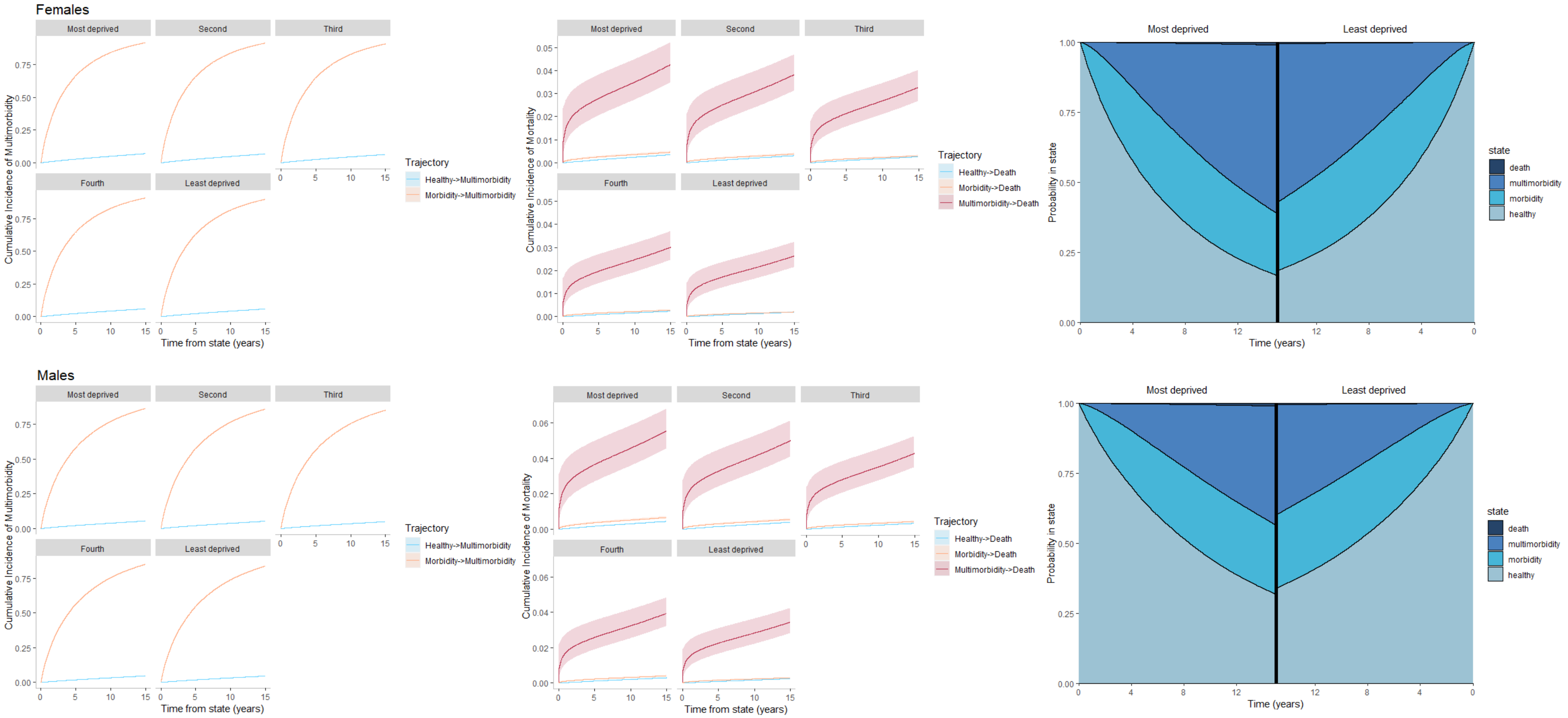

Supplementary Figure 6: Comparison of the cumulative incidence of multimorbidity and mortality between a 50 year old living in one of the most deprived areas of Wales and a 50 year old living in one of the least deprived areas of Wales by sex.

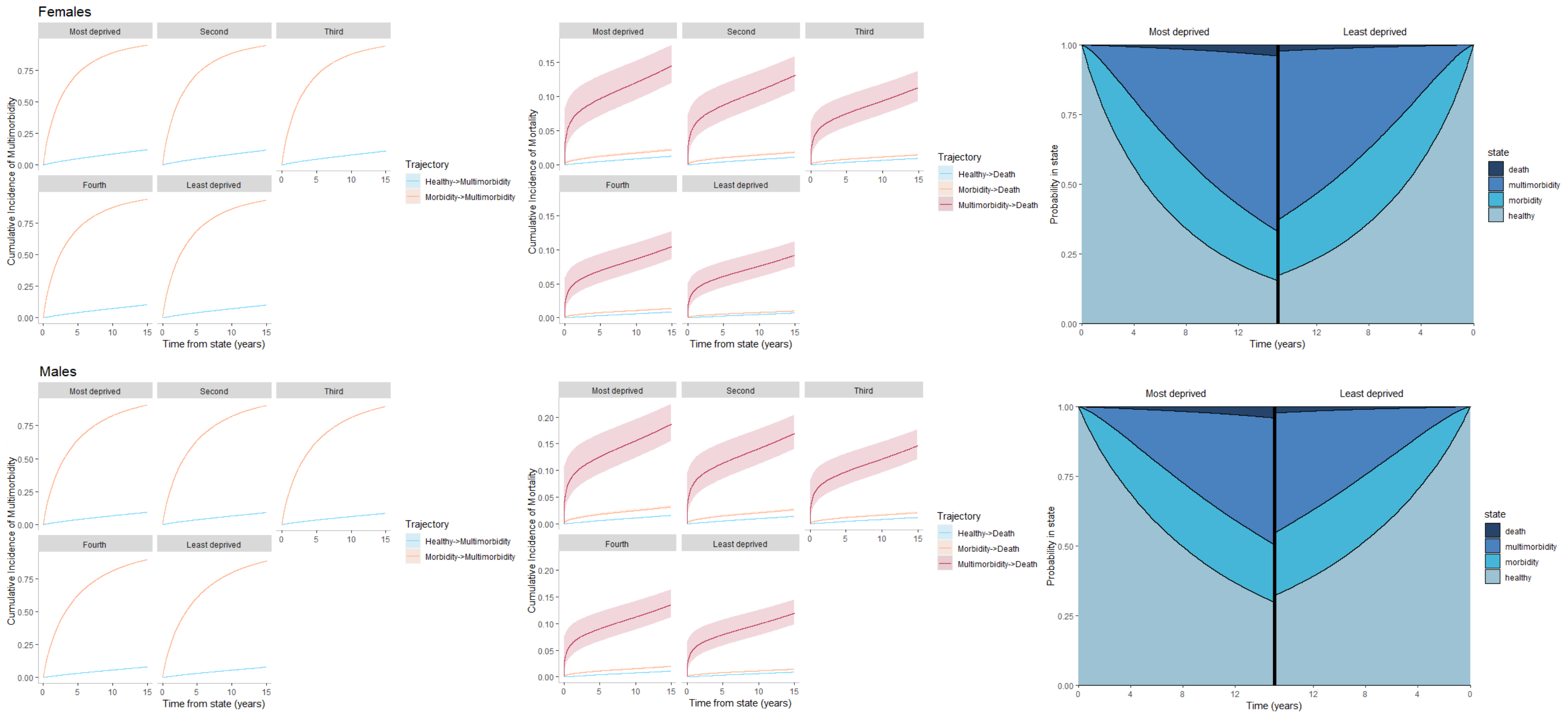

Supplementary Figure 7: Comparison of the cumulative incidence of multimorbidity and mortality between a 60 year old living in one of the most deprived areas of Wales and a 60 year old living in one of the least deprived areas of Wales by sex.

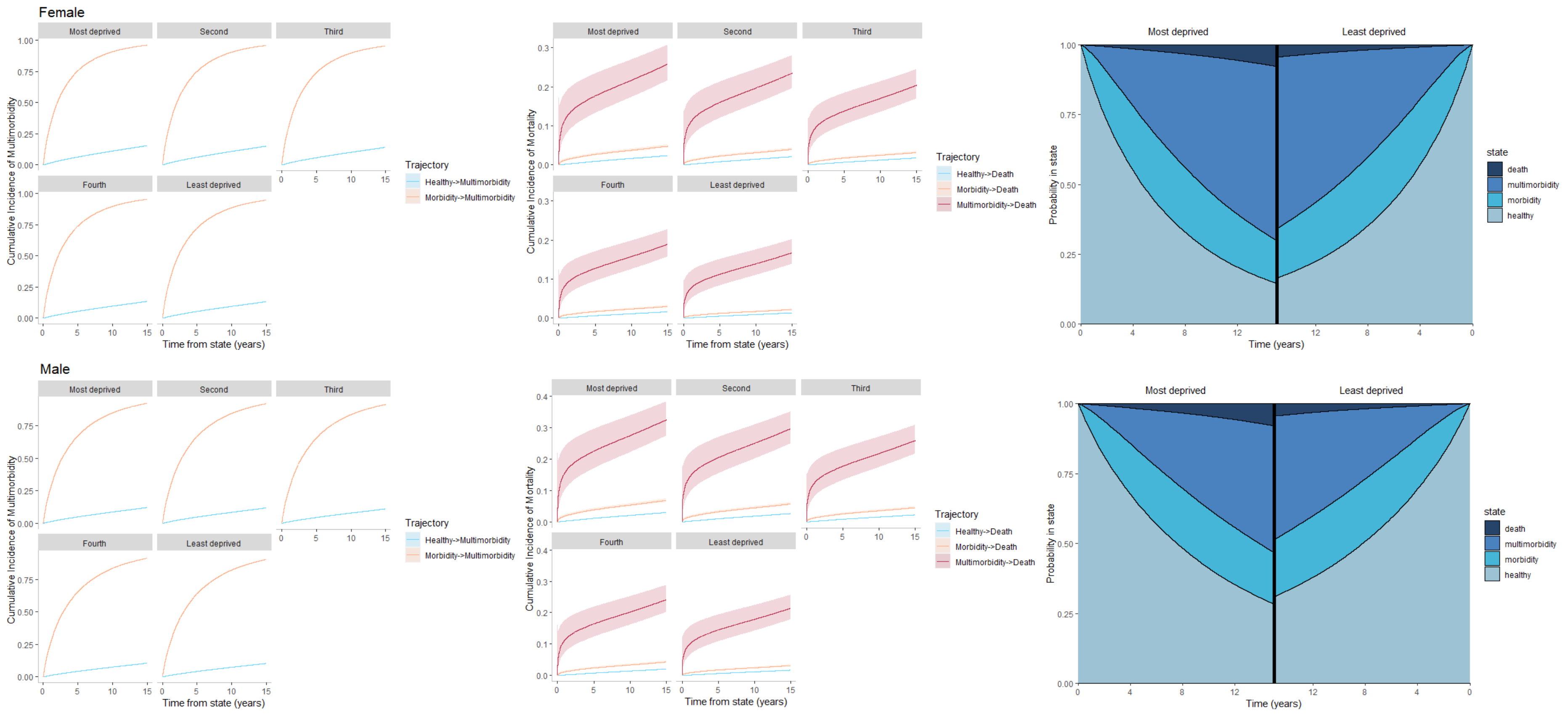

Supplement: Supplementary Tables and Figures [file mmc1.pdf]
